# Impaired phosphocreatine metabolism in white adipocytes promotes inflammation

**DOI:** 10.1038/s42255-022-00525-9

**Published:** 2022-02-14

**Authors:** Salwan Maqdasy, Simon Lecoutre, Gianluca Renzi, Scott Frendo-Cumbo, David Rizo-Roca, Thomas Moritz, Marta Juvany, Ondrej Hodek, Hui Gao, Morgane Couchet, Michael Witting, Alastair Kerr, Martin O. Bergo, Robin P. Choudhury, Myriam Aouadi, Juleen R. Zierath, Anna Krook, Niklas Mejhert, Mikael Rydén

**Affiliations:** 1 Department of Medicine (H7), Karolinska Institutet, Karolinska University Hospital Huddinge, Huddinge, Sweden; 2 CHU Clermont-Ferrand, Service d’endocrinologie, diabétologie et maladies métaboliques, Clermont-Ferrand, France; 3 Laboratoire GReD, Université Clermont Auvergne, Faculté de Médecine, Clermont Ferrand, France; 4 Department of Molecular Medicine and Surgery, Karolinska Institutet, Stockholm, Sweden; 5 Swedish Metabolomics Centre, Department of Forest Genetics and Plant Physiology, Swedish University of Agricultural Sciences, Umeå, Sweden; 6 The NovoNordisk Foundation Centre for Basic Metabolic Research, Faculty of Health and Medical Sciences, University of Copenhagen, Copenhagen, Denmark; 7 Department of Biosciences and Nutrition, Karolinska Institutet, Huddinge, Sweden; 8 Metabolomics and proteomics core (MPC), Helmholtz Zentrum München, Neuherberg, Germany; 9 Research Unit Analytical BioGeoChemistry, Helmholtz Zentrum München, Neuherberg, Germany; 10 Chair of Analytical Food Chemistry, TUM School of Life Sciences, Freising, Germany; 11 Department of Biosciences and Nutrition, Karolinska Comprehensive Cancer Center, Karolinska Institutet, Huddinge, Sweden; 12 Radcliffe Department of Medicine, University of Oxford, Oxford, UK; 13 Department of Physiology and Pharmacology, Karolinska Institutet, Stockholm, Sweden

**Keywords:** Obesity, Mechanisms of disease, Fat metabolism

## Abstract

The mechanisms promoting disturbed white adipocyte function in obesity remain largely unclear. Herein, we integrate white adipose tissue (WAT) metabolomic and transcriptomic data from clinical cohorts and find that the WAT phosphocreatine/creatine ratio is increased and creatine kinase-B expression and activity is decreased in the obese state. In human in vitro and murine in vivo models, we demonstrate that decreased phosphocreatine metabolism in white adipocytes alters adenosine monophosphate-activated protein kinase activity via effects on adenosine triphosphate/adenosine diphosphate levels, independently of WAT beigeing. This disturbance promotes a pro-inflammatory profile characterized, in part, by increased chemokine (C-C motif) ligand 2 (CCL2) production. These data suggest that the phosphocreatine/creatine system links cellular energy shuttling with pro-inflammatory responses in human and murine white adipocytes. Our findings provide unexpected perspectives on the mechanisms driving WAT inflammation in obesity and may present avenues to target adipocyte dysfunction.

## Abstract (web summary)

Maqdasy, Lecoutre et al. show that increased an phosphocreatine/creatine ratio in white adipocytes drives changes in AMP-activated protein kinase activity and promotes white adipocyte inflammation during obesity.

## Main

Obesity is characterized by excess white adipose tissue (WAT) mass and is linked to complications such as insulin resistance, type 2 diabetes, cardiovascular disease and many common cancers^1,2^. At the white adipocyte level, obesity perturbs multiple processes, including gene transcription and metabolic pathways and promotes a pro-inflammatory phenotype, alterations which together link obesity to many of its sequelae^3–6^. However, the mediators driving these changes in fat cells remain unclear.

Studies in other tissues have shown that polar metabolites exert multiple effects on cell function, ranging from epigenetic changes, receptor binding and activation, to altered bioenergetic states^7–10^. In addition, studies in immune cells demonstrate that alterations in intracellular metabolism are tightly coupled to inflammatory status^11,12^. Collectively, this demonstrates that metabolites can coordinate multiple cellular processes and that disturbances in their levels induce distinct changes in tissue function. Characterization of WAT metabolites in the obese and insulin-resistant states can, therefore, provide insights into the mechanisms that regulate tissue dysfunction^13^. Despite this, the metabolic changes that characterize human obese WAT and how these impact on white adipocyte function and clinical phenotypes are unclear.

To identify pathways disturbed in conditions of excess fat mass, we performed unbiased metabolomic and transcriptomic analyses of human WAT and identified that phosphocreatine/creatine metabolism is perturbed in obesity. In white adipocytes, alterations in this energy shuttling system result in changes in adenosine triphosphate (ATP)/adenosine diphosphate (ADP) levels, which attenuate adenosine monophosphate-activated protein kinase (AMPK) activity, thereby promoting a pro-inflammatory profile. Together with recent studies in beige/brown adipocytes^14–17^ and macrophages^18^, our findings highlight the unique cell type-selective role of the phosphocreatine/creatine pathway and its importance in the regulation of obesity-accelerated inflammation in WAT.

## Results

### Obesity perturbs phosphocreatine metabolism in human WAT

**Fig. 1:**
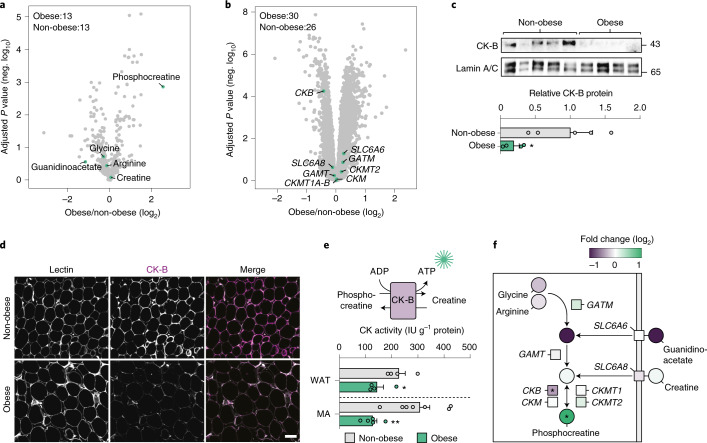
Obesity is associated with altered phosphocreatine/creatine metabolism in human WAT. **a**, Polar metabolites in subcutaneous WAT of obese (*n* = 13) and non-obese (NO, *n* = 13) subjects (cohort 1) highlighting metabolites in the phosphocreatine/creatine pathway (green dots). Data are represented in a volcano plot with fold changes (log_2_) and adjusted *P* values (negative (neg.) log_10_). Statistics were calculated by Welch’s two-sample *t*-test followed by false discovery rate (FDR) correction for multiple comparisons (*q* value), according to standard procedures from Metabolon. **b**, Expression of genes encoding proteins in the phosphocreatine/creatine pathway in subcutaneous WAT of obese (*n* = 30) and non-obese (*n* = 26) women (cohort 2). Data are represented in a volcano plot with fold changes (log_2_) and adjusted *P* values (neg. log_10_) calculated using Limma (linear models for microarray and RNA-seq analysis). **c**, Western blot analysis of CK-B in subcutaneous WAT of obese (*n* = 4) and non-obese (*n* = 5) subjects. Lamin A/C was used as a loading control. *P = 0.036 by Student’s two-sided *t*-test. **d**, Representative immunofluorescence microphotographs of subcutaneous WAT from obese (*n* = 3) and non-obese (*n* = 3) subjects. Sections were stained with *Lens culiniaris* agglutinin (Lectin) and antibodies targeting CK-B. Scale bar, 50 μm. **e**, Creatine kinase activity was measured in total subcutaneous WAT (*n* = 4 from obese and *n* = 4 non-obese subjects) as well as isolated mature adipocytes (*n* = 5 from obese and *n* = 7 non-obese subjects). As illustrated in the upper panel, the creatine kinase activity measured in this assay represents the reverse reaction after addition of ADP where ATP is generated from phosphocreatine. Lower CK-B activity is expected to result in attenuated ATP levels. *P = 0.05; **P = 0.003 by Student’s two-sided *t*-test. **f**, Overview of the phosphocreatine/creatine pathway highlighting the alterations in human subcutaneous WAT linked to obesity. Data are represented as fold changes obese versus non-obese (log_2_). *Significant. In **c** and **e**, data are shown as mean ± s.e.m. CK-B, cytokine B; MA, mature adipocytes. Source data

To identify polar metabolites altered by excess fat mass, we performed an untargeted metabolomic analysis of subcutaneous abdominal WAT obtained from adult women with (*n* = 13) or without (*n* = 13) obesity (cohort 1, described in ref. ^19^ and Supplementary Table 1, Trial registration no. NCT01727245). Among all detected metabolites (*n* = 310), phosphocreatine displayed the highest fold-enrichment in the obese versus the non-obese state (Fig. 1a). Phosphocreatine and ADP are generated from creatine and ATP via specific creatine kinases (CK-MTs, CK-B and CK-M, forward reaction) where CK-B/CK-M can also catalyse the dephosphorylation back to creatine and ATP (reverse reaction)^16^. Cellular creatine can either be taken up directly via the plasma membrane transporter SLC6A8, or be synthesized de novo from the precursor guanidinoacetate, which in turn can be taken up by SLC6A6 or synthesized from glycine and arginine via the mitochondrial glycine amidinotransferase (GATM)^15,20^. Guanidinoacetate is converted to creatine through guanidinoacetate *N*-methyltransferase (GAMT).

**Extended Data Fig. 1:**
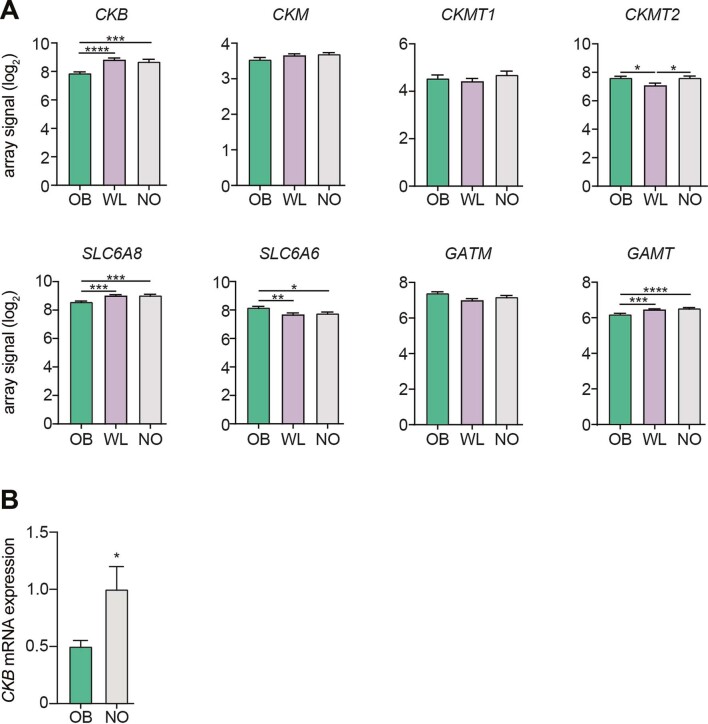
*CKB* expression is regulated by obesity in both women and men. **a**. Expression levels of genes encoding proteins in phosphocreatine/creatine metabolism in cohort 3 consisting of 15 women before (OB) and two years after weight loss (WL) induced by bariatric surgery. The WL group was matched by age and body mass index to a group of 15 never-obese subjects (NO). Data are shown as mean ± SEM. Data were analyzed by one-way ANOVA. Overall p-values were <0.0001 for *CKB*, *SLC6A8* and *GAMT*, 0.010 for *CKMT2* and 0.0023 for *SLC6A6*. Results from Tukey’s post-hoc test are indicated by *p < 0.05, **p < 0.01, ***p < 0.001 and ****p < 0.0001, respectively. **b**. White adipose tissue expression levels of *CKB* determined by qPCR in samples obtained from 18 obese and 17 non-obese men (cohort 4). Data are shown as mean ± SEM. *p = 0.021 by Student’s two-sided t-test.

To determine if the alterations in phosphocreatine metabolism were reflected at the gene expression level, we analysed transcriptomic data of subcutaneous abdominal WAT from women with (*n* = 30) or without (*n* = 26) obesity (cohort 2, described in ref. ^21^ and Supplementary Table 1). We found that *CKB* displayed markedly lower expression in the obese state (Fig. 1b). As displayed in Extended Data Fig. 1a-b, this was confirmed in two independent datasets, where *CKB* levels were lower in 15 obese versus 15 never-obese women (cohort 3, described in ref. ^22^ and Supplementary Table 1, Trial registration no. NCT01785134) and 18 obese versus 17 non-obese men (cohort 4, Supplementary Table 1, Trial registration no. NCT01727245). In cohort 3, *CKB* expression levels were completely normalized in the obese subjects following weight loss induced by bariatric surgery (Extended Data Fig. 1a). Western blot and immunofluorescence analyses confirmed that CK-B levels were lower in WAT of obese versus non-obese subjects (Fig. 1c,d), which was mirrored by attenuated CK activity in lysates obtained from both intact WAT and isolated mature fat cells of non-obese and obese women (Fig. 1e). Altogether, our systematic analyses of metabolomic and transcriptomic data, as well as protein levels and creatine kinase activity measures in human WAT, demonstrate that phosphocreatine abundance is increased and that CK-B expression/activity is decreased in obesity (Fig. 1f).

### Adipocyte *CKB* depletion induces a pro-inflammatory response

**Fig. 2:**
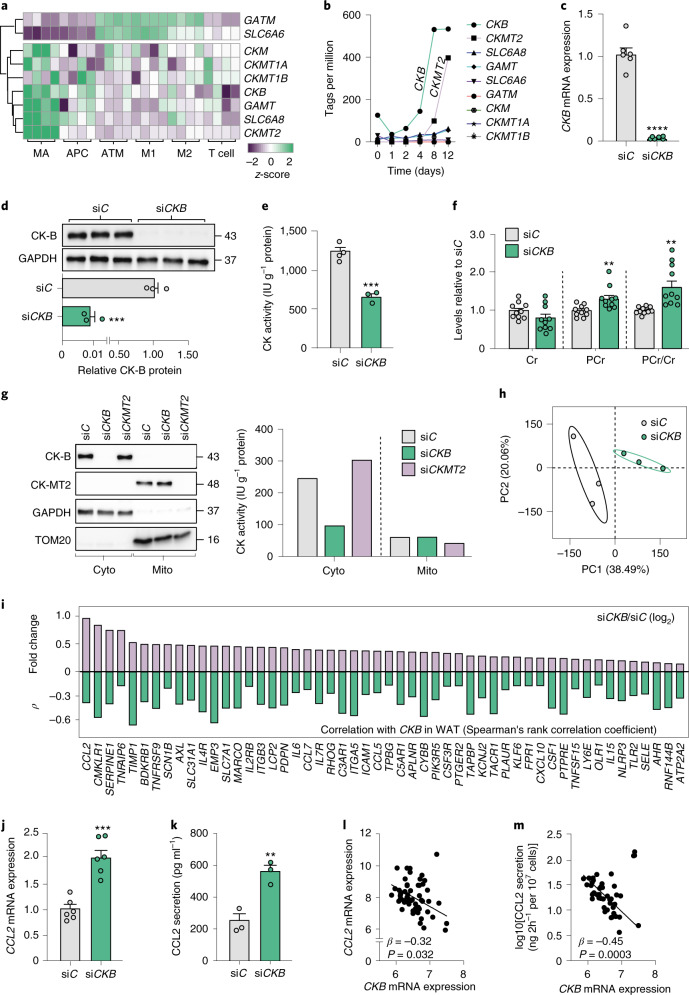
*CKB* silencing induces a pro-inflammatory profile in human white adipocytes. **a**, Expression of indicated genes in cells of human subcutaneous WAT. Results are displayed as *z*-scores. **b**, Expression of indicated genes during adipogenesis. **c***, CKB* mRNA levels in human adipocytes (six replicates per condition, repeated three times) transfected with non-silencing (si*C*) or *CKB*-targeting (si*CKB*) oligonucleotides. ****P < 0.0001. **d**, Western blot displaying CK-B and glyceraldehyde 3-phosphate dehydrogenase (GAPDH) in human adipocytes (three replicates per condition, repeated three times) transfected with si*C* or si*CKB*. ***P = 0.0002. **e**, Creatine kinase activity measured in lysates of human adipocytes (three to four replicates per condition, repeated three times) transfected with si*C* or si*CKB*. ***P = 0.0003. **f**, Metabolite levels in human adipocytes (ten replicates per condition, repeated twice) transfected with si*C* or si*CKB*. **P = 0.0093 for phosphocreatine and 0.002 for phosphocreatine/creatine ratio. **g**, Western blot showing the protein levels in mitochondrial and cytoplasmic fractions of human adipocytes transfected with si*C*, si*CKB* or si*CKMT2* (repeated three times). Creatine kinase activity was determined in paired lysates as indicated in the right panel from one experiment. **h**, Principal component analysis based on microarray data from human adipocytes (three replicates per condition) transfected with si*C* or si*CKB*. Ellipses indicate 95% confidence intervals. **i**, log_2_(fold-change) of genes regulated by si*CKB* in human adipocytes (upper half, from microarray data presented in **h**). For each gene, the association (Spearman’s rank correlation coefficient *ρ* value) with *CKB* expression in WAT transcriptomic data from cohort 2 is shown (lower half). Leading edge genes in the GSEA ‘HALLMARK_INFLAMMATORY _RESPONSE’ pathway identified in Extended Data Fig. 2g are shown. **j***, CCL2* mRNA expression in human adipocytes (six replicates per condition, repeated three times) transfected with si*C* or si*CKB*. ***P = 0.0002. **k**, CCL2 secretion measured by ELISA from human adipocytes (three replicates per condition, repeated three times) transfected with si*C* or si*CKB*. **P = 0.0059. **l**, Correlation between *CCL2* and *CKB* mRNA levels in human WAT from cohort 2. **m**, Correlation between CCL2 WAT secretion and *CKB* mRNA levels in human WAT from cohort 2. In **c**–**f**, **j** and **k**, Student’s two-sided *t*-test was used. In **l** and **m**, standardized *β* and *P* values are shown for multiple regression analysis after BMI correction. Data in **c**–**f**, **j** and **k** are shown as mean ± s.e.m. APC, adipocyte progenitor cells; ATM, adipose tissue macrophages; cyto, cytoplasm; Cr, creatine; M1, M1-macrophages; M2, M2-macrophages; mito, mitochondria, PC, principal component; PCr, phosphocreatine. Source data

**Extended Data Fig. 2:**
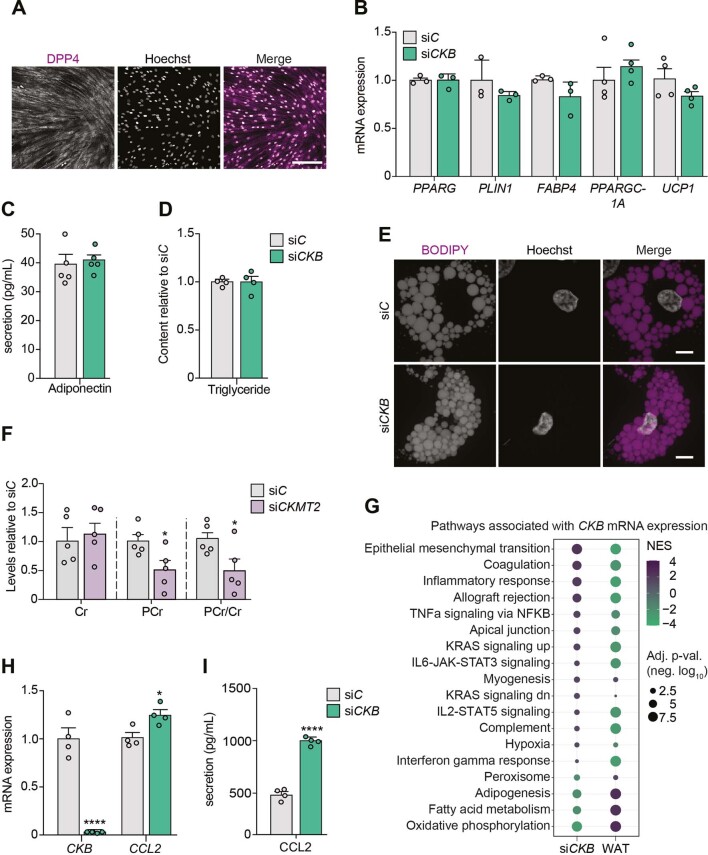
*CKB* depletion does not affect adipogenesis. **a**. Representative immunofluorescence microphotographs of adipocyte progenitors stained with Hoechst and antibodies targeting DPP4. Scale bar=200 μm. **b-e**. *In vitro* differentiated human adipocytes were transfected with scrambled non-silencing (si*C*) or *CKB*-targeting (si*CKB*) (three-four replicates/condition). Effects on gene expression of adipogenic/thermogenic markers (experiment repeated twice) (B), adiponectin secretion (five replicates/condition) (C), cellular triglyceride levels (four replicates/condition) (D) and lipid droplet morphology (from experiments in panel D) (E) were determined. Scale bar in panel E = 10 μm. Data in bar charts are shown as mean ± SEM. **f**. PCr and Cr levels in *in vitro* differentiated human adipocytes transfected with scrambled non-silencing (si*C*) or *CKMT2*-targeting (si*CKMT2*) oligonucleotides (n = 5 replicates/group). Data are shown as mean ± SEM. *p = 0.024 for PCr and 0.045 for PCr/Cr, respectively by Student’s two-sided t-test. **g**. Gene set enrichment analysis (GSEA) of genes correlating with *CKB* expression in human WAT and regulated by si*CKB* transfection in human *in vitro* differentiated adipocytes. Hallmark gene sets from MsigDB were used to calculate the enrichment. Details on the analyses are found in the Methods section. The data are presented as dot plots. The color of the dot indicates the normalized enrichment score (NES) for each pathway. The size of the dot represents the negative log_10_ adjusted p-value for the enrichment of each pathway. **h**. *CKB* and *CCL2 mRNA* levels in *in vitro* differentiated human adipocytes, derived from a female donor, transfected with si*C*) or si*CKB* (four replicates/condition). Data are shown as mean ± SEM. *p = 0.024, ****p < 0.0001 by Student’s two-sided t-test. **i**. Same experiment as in panel H but displaying CCL2 secretion (four replicates/condition). Data are shown as mean ± SEM.****p < 0.0001 by Student’s two-sided t-test. **Abbreviations:** Adj. p-val.=adjusted p-value, Cr=creatine, NES = normalized enrichment score, PCr=phosphocreatine, WAT = white adipose tissue.

To dissect which adipose resident cells contribute to the generation of phosphocreatine, we analysed transcriptomic data from fractionated human WAT^23^. This revealed that mature adipocytes predominatly express *CKB*, *CKMT2*, *GAMT* and *SLC6A8*, while *GATM* and *SLC6A6* are primarily expressed in adipose tissue macrophages (Fig. 2a). As a result, human white adipocytes express most of the genes involved in phosphocreatine/creatine metabolism, an observation that is supported by recent data showing that murine white adipocytes supply breast cancer cells with creatine metabolites^20^. We then determined whether human adipose-derived multipotent mesenchymal DPP4^+^ progenitors (Extended Data Fig. 2a) differentiated into adipocytes are a suitable model system to study this pathway in vitro. Quantitative analyses of expression levels during differentiation showed that the adipocyte-enriched phosphocreatine/creatine metabolism genes, in particular *CKB* and *CKMT2*, were highly expressed in the differentiated state (Fig. 2b) allowing us to study the effects of phosphocreatine/creatine perturbations on white adipocyte phenotype in vitro.

As *CKB* expression was decreased in the obese state, we performed *CKB* knockdown experiments in human adipocytes. Targeting *CKB* by RNA interference (RNAi) resulted in a marked reduction in *CKB* messenger RNA (mRNA) abundance (Fig. 2c), CK-B protein levels (Fig. 2d) and attenuated CK activity (Fig. 2e). In addition, *CKB* silencing increased phosphocreatine abundance and the phosphocreatine/creatine ratio (Fig. 2f) without affecting creatine levels (Fig. 2f). *CKB* knockdown did not alter adipogenesis (Extended Data Fig. 2b–e) or thermogenic gene expression (Extended Data Fig. 2b). As CK-B localizes to both cytosol and mitochondria in brown adipocytes^17^, we next determined the subcellular distribution of CK-B in human white adipocytes. Fractionation experiments revealed that CK-B is cytosolic, and that *CKB* depletion resulted in decreased CK-B protein and CK activity specifically in this fraction (Fig. 2g). In contrast, *CKMT2* silencing resulted in reduced protein abundance and CK activity in mitochondria but not in the cytosol (Fig. 2g). In addition, *CKMT2* depletion resulted in decreased phosphocreatine levels and phospocreatine/creatine ratio without altering creatine abundance (Extended Data Fig. 2f). From these results in human white adipocytes, we conclude that (1) *CKB* is the most highly expressed CK, (2) CK-B resides in the cytosol and (3) CK-B downregulation results in increased cellular phosphocreatine/creatine ratio.

Given that alterations in bioenergetic states are associated with marked transcriptional changes^24^, we next determined the effects of *CKB* depletion on global gene transcription in human white adipocytes. A principal component analysis showed that *CKB* knockdown cells were clearly separated from control-transfected adipocytes (Fig. 2h). To identify obesity-perturbed pathways affected by reduced *CKB* levels, we integrated these in vitro transcriptomic data with genes co-expressed with *CKB* in WAT of the 56 non-obese and obese women in cohort 2. This approach revealed that pro-inflammatory pathways were upregulated by si*CKB* in vitro and linked to low *CKB* expression in vivo (Extended Data Fig. 2g). Analyses of individual genes within the pro-inflammatory pathways showed that *CCL2* (encoding the chemokine Monocyte Chemoattractant Protein-1) displayed the largest fold-change upon si*CKB* (Fig. 2i). As adipocyte CCL2 secretion is increased in obesity and promotes WAT inflammation in both humans and animal models^25–28^, we focused our subsequent analyses on this chemokine. We validated the microarray findings by quantitative PCR (qPCR; Fig. 2j) and ELISA (Fig. 2k) and thus confirmed that *CKB* depletion upregulated both *CCL2* gene expression and CCL2 protein secretion. The data above were obtained in cells from a male donor. However, similar effects were observed in differentitated adipocytes derived from a female donor (Extended Data Fig. 2h,i). The clinical relevance of these findings was further supported by multiple regression analyses in cohort 2 where *CCL2* mRNA levels (Fig. 2l), CCL2 secretion (Fig. 2m) and high sensitivity CRP levels (a measure of inflammation at the whole-body level, standardized *β* = −0.40, *P* = 0.0088, graph not shown) were negatively associated with *CKB* expression after correction for body mass index (BMI).

### *CKB* depletion regulates CCL2 through mitochondrial activity

**Fig. 3:**
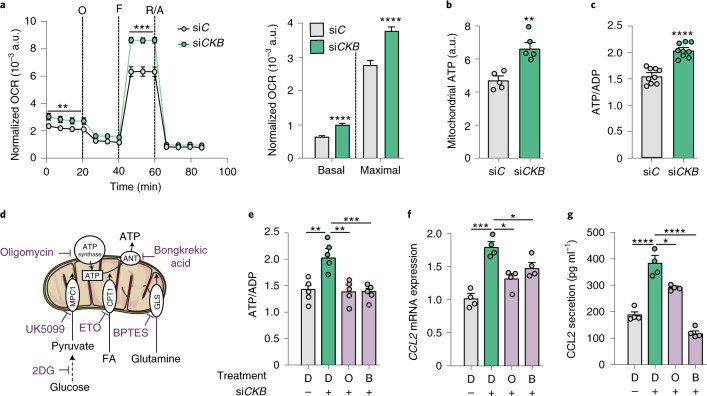
Adipocyte CK-B regulates *CCL2* expression by modulating mitochondrial energy production. **a**, Normalized OCR determined in human adipocytes transfected with scrambled non-silencing (si*C*) or *CKB*-targeting (si*CKB*) oligonucleotides (12 replicates per condition, repeated three times). Non-mitochondrial respiration was substracted from the basal and maximal respiration is represented in the bar chart. **P = 0.0059, ***P = 0.0008, ****P < 0.0001 (both basal and maximal respiration). O, Oligomycin; F, FCCP; R/A, rotenone/antimycin. **b**, Mitochondrial ATP production based on Seahorse data in human adipocytes transfected with si*C* or si*CKB* (*n* = five replicates per condition, repeated twice). **P = 0.0078. **c**, ATP/ADP ratio measured by bioluminescence in human adipocytes transfected with si*C* or si*CKB* (nine replicates per condition, repeated twice). ****P < 0.0001. **d**, Schematic representation of drugs targeting mitochondrial ATP production and/or substrate usage. **e**, Effect of the mitochondrial inhibitors 1 μM oligomycin (O) or 1 μM bongkrekic acid (B) for 24 h on ATP/ADP ratio in human adipocytes transfected with si*C* or si*CKB*. Control wells were treated with dimethyl sulfoxide (DMSO) (D) (five replicates per condition, repeated twice). Overall *P* = 0.043. **f**, Same experimental setup as **e** but displaying *CCL2* mRNA expression (four replicates per condition, repeated twice). Overall *P* = 0.0003. **g**, Same experimental setup as in **e** but displaying CCL2 secretion (pg ml^−1^) detected by ELISA (four replicates per condition, repeated twice). Overall *P* < 0.0001. Data were analysed by Student’s two-sided *t*-test in **a**–**c** and by one-way ANOVA in **e**–**g** (Tukey’s post-hoc tests indicated by *P < 0.05, **P < 0.01, ***P < 0.001, ****P < 0.0001). Data are shown as mean ± s.e.m. 2DG, 2-deoxy-D-glucose; ANT,adenine nucleotide translocase; a.u., arbitray unit; CPT1, carnitine palmitoyl transferase 1; FA, fatty acid; GLS, glutaminase; MPC1, mitochondrial pyruvate carrier 1.

**Extended Data Fig. 3:**
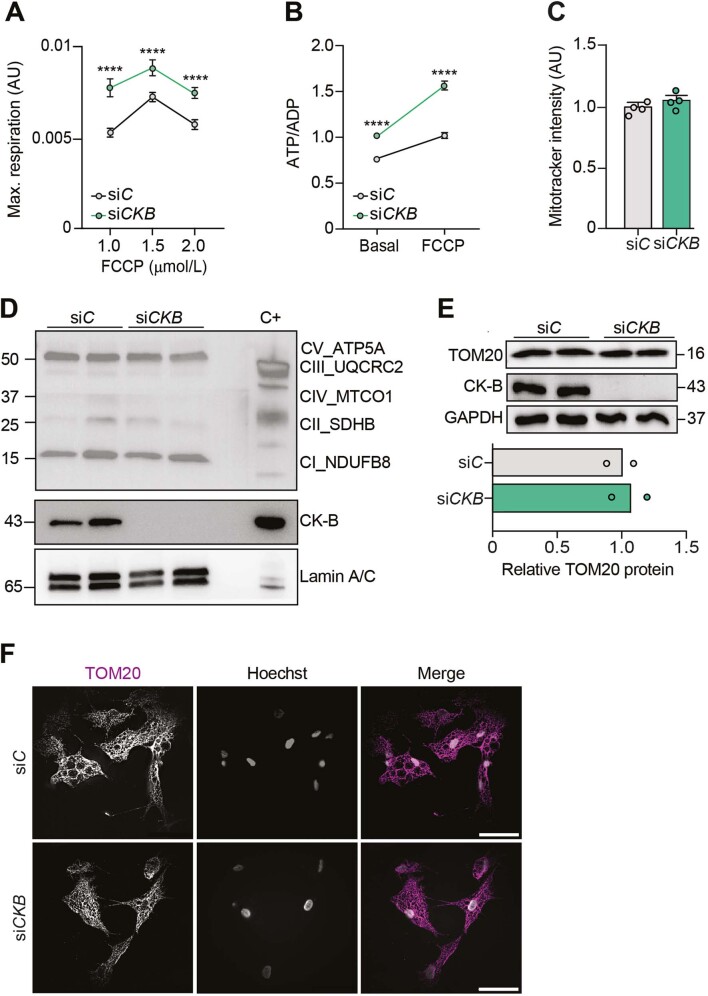
*CKB* depletion does not affect mitochondrial content and morphology. **a**. Maximal respiration of *in vitro* differentiated human adipocytes determined by Seahorse Mito stress analysis using 1-2 μmol/L of FCCP, after transfection of the cells with si*C* or si*CKB* (12 replicates/condition). Data are shown as mean ± SEM. ****p < 0.0001 by Student’s two-sided t-test. **b**. ATP/ADP levels in *in vitro* differentiated human adipocytes transfected with si*C* or si*CKB* after 1 hour of incubation with 1.5 μmol/L of FCCP (eight replicates per condition). Data are shown as mean ± SEM. ****p < 0.0001 by Student’s two-sided t-test. **c**. Quantification of Mitotracker^TM^ red fluorescence intensity in *in vitro* differentiated human adipocytes transfected with si*C* or si*CKB* (four replicates/condition, repeated twice). Data are shown as mean ± SEM. **d-e**. *In vitro* differentiated human adipocytes were transfected with si*C* or si*CKB* (two replicates per blot). Protein levels of OXPHOS components (D) and TOM20 (mean values) (E) were determined together with CK-B and GAPDH by western blot to estimate mitochondrial abundance. **f**. Representative immunofluorescence microphotographs of *in vitro* differentiated adipocytes transfected with si*C* or si*CKB* and stained with antibodies targeting TOM20 and Hoechst. Scale bar=50 μm. **Abbreviations:** ATP5A = Mitochondrial membrane ATP Synthase, AU = arbitrary units, FCCP = Carbonyl cyanide-p-trifluoromethoxyphenylhydrazone, C + = positive control, mitochondrial extract from rat heart supplied with the antibody as a positive control, MTCO1 = Mitochondrially Encoded Cytochrome C Oxidase I, NDUFB8 = NADH:Ubiquinone Oxidoreductase Subunit B8, SDHB = Succinate dehydrogenase B, UQCRC2 = Cytochrome b-c1 complex subunit 2, mitochondrial. Source data

Creatine metabolism regulates the bioenergetic state of cells^29^ and the latter is linked to inflammation and immune cell activation^30–33^. As a result, we hypothesized that the association between *CKB* depletion and CCL2 production is mediated by altered energy balance. We tested this by performing functional analyses of mitochondrial activity. Following carbonyl cyanide-*p*-trifluoromethoxyphenylhydrazone (FCCP)-titration experiments, we found that the oxygen consumption rate (OCR) was increased following *CKB* knockdown (Fig. 3a and Extended Data Fig. 3a); this effect was accompanied by increased mitochondrial ATP production and cellular ATP/ADP ratio (Fig. 3b,c and Extended Data Fig. 3b), while mitochondrial abundance and morphology remained unchanged (Extended Data Fig. 3c–f). To determine if the perturbations in mitochondrial ATP production observed on *CKB* depletion are required to upregulate *CCL2* expression, we incubated si*C*- and si*CKB*-transfected adipocytes with or without oligomycin (an ATP synthase inhibitor) or bongkrekic acid (an adenine nucleotide translocase inhibitor) (Fig. 3d). Both inhibitors prevented the increase in ATP/ADP ratio (Fig. 3e) and CCL2 expression/secretion induced by *CKB* RNAi (Fig. 3f,g), suggesting that changes in ATP/ADP ratio are required for mediating the effects of *CKB* depletion on *CCL2* expression.

### CCL2 induction upon *CKB* depletion requires glucose usage

**Fig. 4:**
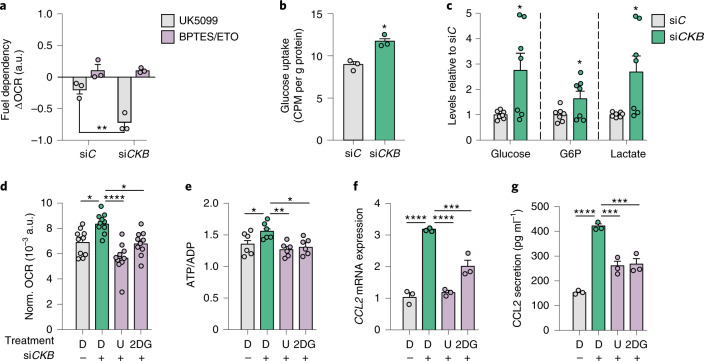
Mitochondrial activation upon *CKB* downregulation is driven by increased glycolytic activity and glucose uptake. **a**, Delta OCR determined by mitochondrial fuel flex test using drugs targeting mitochondrial substrate usage (glycolysis, fatty acid oxidation and glutaminolysis) in human adipocytes transfected with si*C* or si*CKB* (three replicates per condition, repeated twice). Overall *P* = 0.0099. **b**, Glucose uptake levels in human adipocytes transfected with si*C* or si*CKB* (three replicates per condition, repeated twice). *P = 0.023. **c**, Intracellular levels of glucose, glucose-6-phosphate and lactate in human adipocytes transfected with si*C* or si*CKB* (seven replicates per condition). *P = 0.02, 0.049 and 0.02, respectively (from left to right). **d**. Normalized OCR in human adipocytes transfected with si*C* or si*CKB* and incubated with 0.1 mmol l^−1^ 2DG or 10 μmol l^−1^ of UK5099 (U) or DMSO (D) for 24 h (ten replicates per condition). Overall *P* < 0.0001. **e**, Same experimental setup as in **d** but displaying the effects on ATP/ADP ratio (six replicates per condition). Overall *P* = 0.0048. **f**, Same experimental setup as in **d** but displaying the effects on *CCL2* mRNA expression (three replicates per condition, repeated twice). Overall *P* < 0.0001. **g**, Same experimental setup as in **d** but displaying the effects on CCL2 secretion (pg ml^−1^) (three replicates per condition, repeated twice). Overall *P* < 0.0001. Data were analysed by Student’s two-sided *t*-test in **b** and **c**, by one-way ANOVA in **d**–**g** and by two-way ANOVA in **a** (Tukey’s post-hoc tests indicated by *P < 0.05, **P < 0.01, ***P < 0.001, ****P < 0.0001). Data are shown as mean ± s.e.m. CPM, counts per minute; G6P, glucose-6-phosphate; norm., normalized.

**Extended Data Fig. 4:**
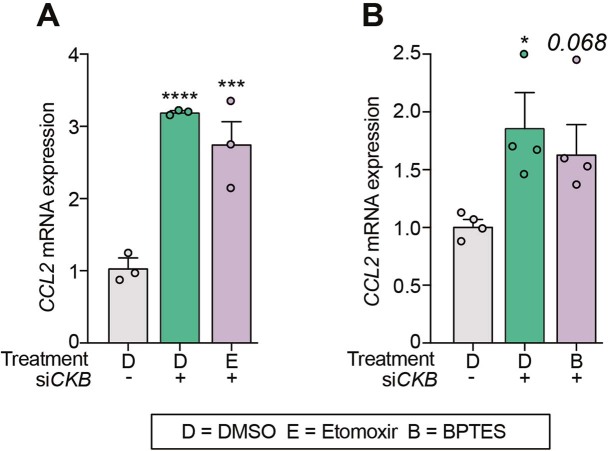
White adipocyte mitochondrial hyperactivity following *CKB* silencing is not driven by fatty acid oxidation or glutaminolysis. **a-b**. *CCL2* expression in *in vitro* differentiated human adipocytes transfected with either si*C* or si*CKB*, and treated with 3 μmol/L Etomoxir (fatty acid oxidation inhibitor) (three replicates/condition) (A) or 10 μmol/L BPTES (glutaminolysis inhibitor) (four replicates per condition) (B). Control cells were treated with DMSO. Data are shown as mean ± SEM. Overall p < 0.0001 for panel A and 0.028 for panel B by one-way ANOVA. Results from Tukey’s post-hoc test are indicated by *p < 0.05, ***<0.001 and ****p < 0.0001, respectively. **Abbreviations:** BPTES = Bis-2-(5-phenylacetamido-1,3,4-thiadiazol-2-yl)ethyl sulfide, DMSO = dimethyl sulfoxide.

We next determined whether the link between the ATP/ADP ratio and *CCL2* expression upon *CKB* knockdown was dependent on a specific macronutrient. We studied acute changes in OCR of control or *CKB*-silenced white adipocytes treated with inhibitors of mitochondrial pyruvate transport (2-cyano-3-(1-phenyl-1H-indol-3-yl)-2-propenoic acid (UK5099)), glutaminase (bis-2-(5-phenylacetamido-1,3,4-thiadiazol-2-yl)ethyl sulfide (BPTES)) or mitochondrial fatty acid translocation (Etomoxir (ETO)) (Fig. 3d). UK5099, not BPTES or ETO, impaired the rise in OCR of *CKB*-depleted white adipocytes (Fig. 4a). This suggests that *CKB* knockdown increases glucose-derived carbon utilization, a notion supported by the observation that glucose uptake and intermediates in the glycolytic pathway (glucose, glucose-6-phosphate and lactate) were increased in *CKB*-depleted cells (Fig. 4b,c). We further tested the link between increased glucose utilization and *CCL2* by comparing the effects of UK5099 and the hexokinase inhibitor 2-deoxy-D-glucose (2DG). Both inhibitors prevented the induction of OCR (Fig. 4d), the rise in the ATP/ADP ratio (Fig. 4e), as well as elevation of *CCL2* mRNA expression and secretion (Fig. 4f,g) upon *CKB* silencing. In contrast, the elevation of *CCL2* expression upon *CKB* silencing was unaltered by ETO and BPTES treatment (Extended Data Fig. 4a,b). Together, these data suggest that the the import of glucose-derived carbons into mitochondria is required for activating inflammatory gene expression upon *CKB* silencing in white adipocytes.

### Reduction in AMPK activity induces *CCL2* expression

**Fig. 5:**
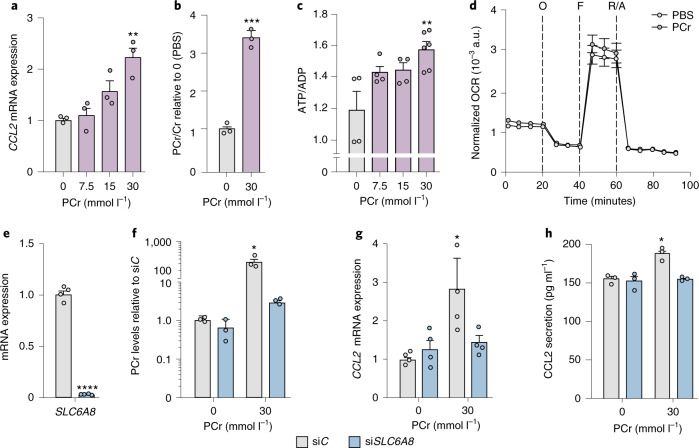
Phosphocreatine is taken up by SLC6A8 in white adipocytes and promotes CCL2 production. **a**, Human adipocytes were incubated with PBS or different concentrations of phosphocreatine for 24 h and effects on *CCL2* mRNA expression were determined (three replicates per condition, repeated twice). Overall *P* = 0.0021. **b**, The ratio of phosphocreatine/creatine in human in vitro differentiated adipocytes incubated with PBS or 30 mmol l^−1^ phosphocreatine for 24 h (three replicates per condition, repeated twice). ***P = 0.0002. **c**, Same experiments as in **a** but displaying the ratio of ATP/ADP ratio (four replicates per condition for 0–15 mmol l^−1^and six replicates per condition for 30 mmol l^−1^, repeated twice). Overall *P* = 0.0068. **d**, Normalized OCR in human adipocytes incubated with 30 mmol l^−1^ phosphocreatine for 24 h (six replicates per condition, repeated twice). **e**, Expression of *SLC6A8* in human adipocytes transfected with scrambled non-silencing (si*C*) or *SLC6A8*-targeting (si*SLC6A8*) oligonucleotides (four replicates per condition, repeated three times). ****P < 0.0001. **f**, Phosphocreatine levels in human adipocytes transfected with si*C* or si*SLC6A8*, and incubated in the presence or absence of 30 mmol/L phosphocreatine for 24 h (three replicates per condition, repeated twice). Overall *P* = 0.0028. **g**, Same experimental setup as in **f** but displaying the effects on *CCL2* mRNA levels (four replicates per condition, repeated twice). Overall *P* = 0.05. **h**, Same experimental setup as in **f** but displaying the effects on CCL2 secretion (three replicates per condition, repeated twice). Overall *P* = 0.05. Data were analysed by Student’s two-sided *t*-test in **b** and **e**, by one-way ANOVA in **a** and **c** and by two-way ANOVA in **f**–**h**. Tukey’s post-hoc tests indicated by *P < 0.05, **P<0.01. Data are shown as mean ± s.e.m.

**Extended Data Fig. 5:**
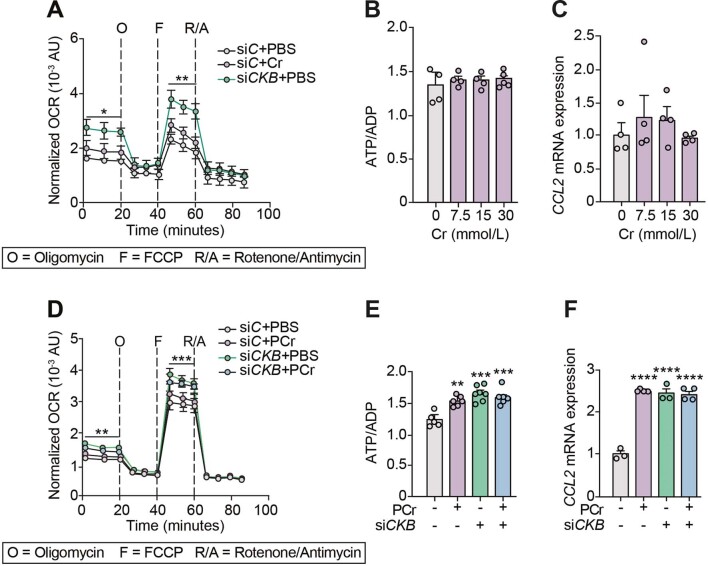
Differential effects of creatine and phosphocreatine in human white adipocytes. **a**. Effect of creatine on OCR by Mito Stress test in *in vitro* human adipocytes transfected with either si*C* or si*CKB* incubated with or without creatine (20 mmol/L) (six replicates/condition) for 24 hours. Data are shown as mean ± SEM. *p = 0.029 and **p = 0.0012 by one-way ANOVA. **b**. ATP/ADP levels in *in vitro* differentiated human adipocytes incubated with creatine (7.5–30 mmol/L) for 24 hours (four replicates/condition). Data are shown as mean ± SEM. **c**. Same experimental setup as panel B but displaying *CCL2* expression (four to five replicates/condition, experiment repeated twice). Data are shown as mean ± SEM. **d**. *In vitro* differentiated human adipocytes transfected with either si*C* or si*CKB* were incubated with or without phosphocreatine (30 mM) for 24 hours and the effects on OCR were determined by Mito Stress test (six replicates/condition, experiment repeated twice). Data are shown as mean ± SEM. p = 0.007 and 0.0004 for basal and maximal respiration by two-way ANOVA. **e**. ATP/ADP levels in *in vitro* differentiated human adipocytes transfected with either si*C* or si*CKB*, incubated with phosphocreatine (30 mmol/L) for 24 hours (si*C* = five replicates, si*C* + PCr=six replicates, si*CKB* = seven replicates, si*CKB* + PCr=six replicates). Data are shown as mean ± SEM. Overall p = 0.0004 by two-way ANOVA. Results from Tukey’s post-hoc test are indicated by **p < 0.01, ***p < 0.001, respectively. **f**. Same experimental setup as panel E but displaying *CCL2* expression (si*C* = three replicates, si*C* + PCr=four replicates, si*CKB* = three replicates, si*CKB* + PCr=four replicates, experiment repeated twice). Data are shown as mean ± SEM. Overall p < 0.0001 by two-way ANOVA. Results from Tukey’s post-hoc test are indicated by ****p < 0.001. **Abbreviations:** AU = arbitrary unit, Cr=creatine, OCR = oxygen consumption rate, PCr=phosphocreatine.

To test whether the effect of *CKB* depletion on mitochondrial activity was triggered by a reduced ability to generate ATP from phosphocreatine and/or an increase in phosphocreatine abundance, we incubated human adipocytes with phosphocreatine or creatine. While incubations with creatine had no effects on OCR, ATP/ADP ratio or *CCL2* expression (Extended Data Fig. 5a–c), the addition of phosphocreatine increased *CCL2* expression (Fig. 5a), intracellular phosphocreatine levels (Fig. 5b) and the ratio of ATP/ADP (Fig. 5c). However, in contrast to *CKB* depletion, phosphocreatine incubation did not affect OCR (Fig. 5d). To determine whether the observed effects were due to phosphocreatine uptake, we silenced *SLC6A8*. This prevented the increase in intracellular phosphocreatine levels as well as *CCL2* expression and secretion following addition of phosphocreatine (Fig. 5e–h). Furthermore, phosphocreatine had no additive effects on OCR, ATP/ADP ratio and *CCL2* expression in si*CKB*-transfected cells (Extended Data Fig. 5d–f). Taken together, our results indicate that the effects of both si*CKB* and phosphocreatine incubations on *CCL2* expression converge on increases in the ATP/ADP ratio, while alterations in mitochondrial activity are only linked to *CKB* depletion.

**Fig. 6:**
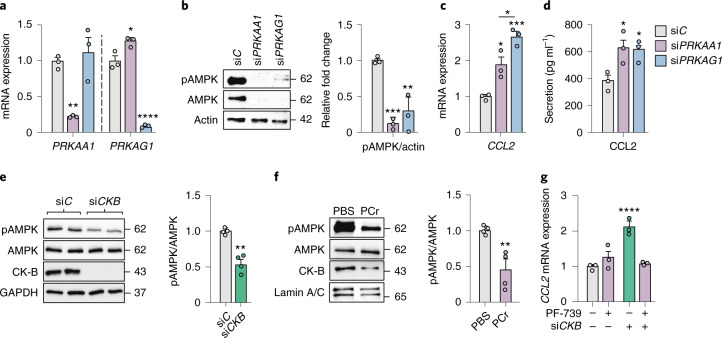
Perturbation in phosphocreatine metabolism is linked to CCL2 production via effects on AMPK activity. **a***, PRKAA1* and *PRKAG1* mRNA expression in human adipocytes transfected with si*PRKAA1*, si*PRKAG1* or si*C* (three replicates per condition, repeated twice). Overall *P* = 0.005 for PRKAA1 and *P* < 0.0001 for PRKAG1. **b**, AMPK, phosphorylated AMPK (pAMPK) and actin levels determined by western blot in human adipocytes transfected with si*PRKAA1*, si*PRKAG1* or si*C*. The left panel shows representative blots and bar graphs to the right show quantifications of the indicated protein levels (three independent experiments). Overall *P* = 0.0013. **c**, Same experimental setup as in **a** but displaying the effects on *CCL2* mRNA levels (three replicates per condition, repeated twice). Overall *P* = 0.0011. **d**, Same experimental setup as in **a** but displaying the effects on CCL2 secretion (three replicates per condition, repeated twice). Overall *P* = 0.0115. **e**, AMPK, pAMPK, CK-B and GAPDH determined by western blot in human adipocytes transfected with si*C* or si*CKB*. The left panel shows representative blots and bar graphs to the right show quantifications of the indicated protein levels (two replicates per condition, repeated two times). **P = 0.0018. **f**, AMPK, pAMPK, CK-B and Lamin A/C determined by western blot in human adipocytes transfected with si*C* or si*CKB*, after incubation for 24 h with phosphocreatine (30 mmol l^−1^) or PBS. The left panel shows representative blots and bar graphs to the right show quantifications of the indicated protein levels (quantifications from four independent expirements). **P = 0.0078. **g***, CCL2* expression in human adipocytes transfected with si*C* or si*CKB*, after incubation with vehicle (DMSO) or PF-739 (5 μmol l^−1^) for 24 h (three replicates per condition, repeated twice). Overall *P* < 0.0001. Data were analysed by Student’s two-sided *t*-test in **e** and **f**, by one-way ANOVA in panels **a**–**d** and two-way ANOVA in **g**. Tukey’s post-hoc tests indicated by *P < 0.05, **P < 0.01, ***P < 0.001, ****P < 0.0001. Data are shown as mean ± s.e.m. Source data

**Extended Data Fig. 6:**
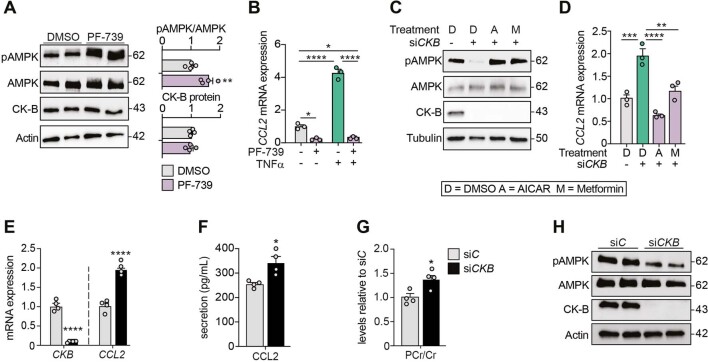
AMPK regulates *CCL2* expression. **a**. *In vitro* differentiated human adipocytes were incubated with DMSO or the AMPK activator PF-739 (n = 4). AMPK, phosphorylated AMPK (pAMPK), CK-B and actin levels were determined by western blot (in duplicates per blot). Left panel shows representative blots and bar graphs and to the right show quantifications of the indicated protein levels (four replicates/condition). Data are shown as mean ± SEM. **p = 0.0087 by Student’s two-sided t-test. **b**. *CCL2* mRNA expression in *in vitro* differentiated human adipocytes following incubation with DMSO (control), TNFα, PF-739 or both TNFα and PF-739 for 24 hours (three replicates/condition). Data are shown as mean ± SEM. Overall p < 0.0001 by two-way ANOVA. Results from Tukey’s post-hoc test are indicated by *p < 0.05, ****p < 0.0001, respectively. **c-d**. AMPK, phosphorylated AMPK (pAMPK), CK-B and tubulin protein levels (C) as well as *CCL2* expression (D) were determined in human *in vitro* differentiated adipocytes transfected with si*C* or si*CKB*, after incubation for 24 hours with DMSO or the AMPK activators AICAR and metformin (3 replicates/condition, experiment repeated twice). Data are shown as mean ± SEM. Overall p = 0.0001 by one-way ANOVA. Results from Tukey’s post-hoc test are indicated by **p < 0.01, ***p < 0.001, ****p < 0.0001, respectively. **e-f**. CCL2 gene expression and secretion were determined in human *in vitro* differentiated adipocytes transfected with si*C* or si*CKB* (four replicates/condition) from a different vendor (Qiagen). Data are shown as mean ± SEM. *p = 0.011, ****p < 0.0001 by Student’s two-sided t-test. **g**. Same experimental setup as panels E-F but displaying phosphocreatine/creatine levels. Data are shown as mean ± SEM. *p = 0.012 by Student’s two-sided t-test. **h**. Same experimental setup as panels E-F but showing representative western blots of phosphorylated AMPK (pAMPK), AMPK, CK-B and actin (two replicates/condition loaded per blot). **Abbreviations:** AICAR = 5-aminoimidazole-4-carboxamide riboside, AU = arbitrary unit, Cr=creatine, PCr=phosphocreatine. Source data

As AMPK senses the ATP/AMP ratio and is a well-established regulator of inflammatory responses (as reviewed in ref. ^34,35^), we hypothesized that decreased AMPK activity could mediate the effects on *CCL2* observed in *CKB*-depleted and phosphocreatine-treated adipocytes. To determine if AMPK impacts inflammation in white adipocytes, we silenced AMPK subunits α1 (encoded by *PRKAA1*) and γ1 (*PRKAG1*) by RNAi. This resulted in reduced levels of AMPK abundance/activity (based on phosphorylation of Threonine 172) and increased CCL2 mRNA expression/secretion (Fig. 6a–d). In contrast, the pan-AMPK activator PF-739 stimulated AMPK activity without affecting the levels of CK-B, and abrogated the induction of *CCL2* transcription by tumour necrosis factor alpha (TNF-α), a canonical positive regulator of inflammation (Extended Data Fig. 6a,b)^36,37^. After establishing a causal link between AMPK and *CCL2* expression in human white adipocytes, we next measured AMPK phosphorylation in cells transfected with si*CKB* or incubated with phosphocreatine. Compared to control cells, AMPK phosphorylation was reduced in both conditions (Fig. 6e,f). To test if the effects of *CKB* depletion on *CCL2* expression depend on reduced AMPK activity, we incubated si*CKB*- and si*C*-transfected cells with and without PF-739. Our data provide evidence that PF-739 abrogated the si*CKB*-induced increase in *CCL2* mRNA expression (Fig. 6g), indicating that AMPK links reduced CK-B to increased CCL2 production. To exclude potential off-target effects, we also tested additional AMPK agonists, including 5-aminoimidazole-4-carboxamide riboside and metformin (Extended Data Fig. 6c,d), as well as another short interfering RNA (siRNA) targeting *CKB* (Extended Data Fig. 6e–h). These experiments reproduced the effects of *CKB* depletion and AMPK activation, further supporting the idea that perturbations in phosphocreatine/creatine metabolism affect *CCL2* expression through reduced AMPK activity in white adipocytes.

### Murine in vivo models corroborate human findings

**Fig. 7:**
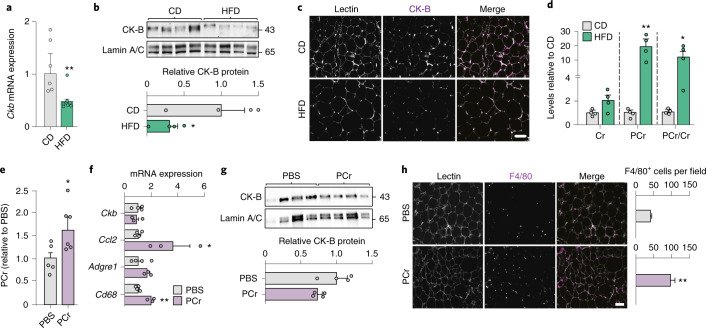
HFD and phosphocreatine injections promote WAT inflammation in vivo. **a**–**d**, Effect of HFD in male mice aged 10–11 weeks on *Ckb* mRNA expression (six mice in CD and eight in HFD group). **P=0.0026. (**a**) and CK-B protein abundance determined by western blot (*n* = 4 per group). *P=0.041. (**b**) or immunofluorescence (**c**) as well as phosphocreatine and creatine levels (*n* = 4 per group). *P=0.019, **P=0.0063. (**d**) in pgWAT. Scale bar, 50 μm. **e**, WAT phosphocreatine levels measured in 12-week-old male mice treated with either PBS or phosphocreatine injections (3 mg g^−1^, intraperitoneally for 7 days, *n* = 5 for PBS-injected and *n* = 6 for phosphocreatine-injected mice). *P = 0.049. **f**, Transcriptional levels of *Ckb* and genes encoding pro-inflammatory markers/factors was determined in pgWAT (*n* = 4 for PBS-injected and *n* = 3 for phosphocreatine-injected animals) of mice described in **e**. *P* = 0.029 for *Ccl2* and 0.0024 for *Cd68*. **g**, CK-B protein levels in pgWAT of mice injected with PBS or phosphocreatine (*n* = 4 per group). **h**, Immunofluorescence of F4/80 (magenta) and *Lens culinaris* agglutinin (Lectin, grey) in pgWAT of phosphocreatine- and PBS-injected mice. The number of F4/80^+^ cells was counted in three to four random fields of pgWAT per mouse in four mice. Scale bar, 50 μm. **P = 0.0017. Data were analysed by Student’s two-sided *t*-test except for **b**, which was analysed by Student’s one-sided *t*-test. Data are shown as mean ± s.e.m. Source data

**Extended Data Fig. 7:**
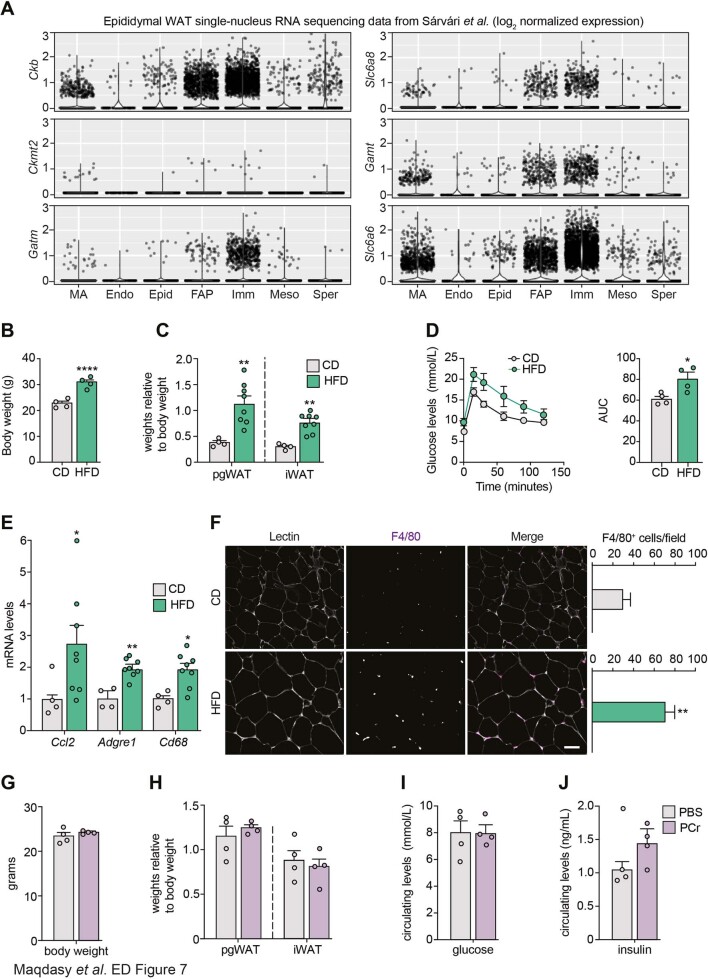
Additional data from murine models. **a**. Expression of phosphocreatine/creatine-related genes in mouse WAT was extracted from single-nucleus RNA sequencing data published by Sárvári *et al*^34^. **b-c**. Wild-type male mice were fed chow (CD) or high fat diet (HFD) for 5 weeks. Effects on body weight (four mice/condition) (B) and pgWAT and iWAT weights (four mice for CD and eight mice for HFD) (C) were determined. Data are shown as mean ± SEM. **p = 0.0034 for pgWAT and 0.0016 for iWAT, respectively, ****p < 0.0001 by Student’s two-sided t-test. **d**. Same experimental setup as in panels B-C but displaying effects on glucose tolerance by intraperitoneal glucose tolerance tests (four mice/group). The area under the curve (AUC) is displayed in the right panel. Data are shown as mean ± SEM. *p = 0.041 by Student’s two-sided t-test. **e**. Same experimental setup as in panels B-C but displaying effects on the expression of genes encoding pro-inflammatory markers/factors in pgWAT (four mice for CD and eight mice for HFD). Data are shown as mean ± SEM. p = 0.049 for *Ccl2*, 0.002 for *Adgre1* and 0.023 for *Cd68*, respectively by Student’s two-sided t-test. **f**. Representative immunofluorescence microphotographs of F4/80 (magenta) and *Lens culinaris* agglutinin (Lectin, gray) in pgWAT of HFD and CD mice. The number of F4/80^+^ cells was counted in 4 random fields (n = 4 mice/group). Scale bar=50 μm. Data are shown as mean ± SEM. **p = 0.0039, by Student’s two-sided t-test. **g-j**. Male mice on chow diet were injected intraperitoneally with PBS or phosphocreatine (3 mg/g) for seven days (n = 4 mice/group). Effects on body weight (G), pgWAT and iWAT weights (H) and circulating glucose (I) as well as insulin (J) levels were determined. Data are shown as mean ± SEM. **Abbreviations:** AUC = area under the curve, CD = chow diet, Endo=endothelial, Epid=Edipidymal, FAP = fibro-adipogenic progenitor, HFD = high fat diet, Imm=immune, iWAT=inguinal WAT, MA = mature adipocytes, Meso=mesothelial, PCr=phosphocreatine, pgWAT=perigonadal WAT, Sper=spermatozoa.

To assess the relevance of our findings in vivo, we analysed published datasets and investigated different mouse models. First, we mined single-nucleus transcriptomics of epididymal WAT^38^ and observed that, similar to humans, *Ckb* is the most highly expressed creatine kinase-encoding gene in white adipocytes (Extended Data Fig. 7a). As obese humans display decreased *CKB* expression and increased phosphocreatine levels, we next determined if a short (five weeks) high fat diet (HFD) regimen recapitulated this phenotype in mice. This intervention length was chosen to determine if alterations in phosphocreatine/creatine metabolism are early events in the development of obesity. As expected, HFD-feeding resulted in increased body weight and fat mass (Extended Data Fig. 7b,c), impaired glucose tolerance (Extended Data Fig. 7d) and WAT inflammation with increased gene expression of *Ccl2*, as well as the macrophage markers *Cd68* and *Adgre1* (F4/80), where the latter was confirmed by immunofluorescence analysis (Extended Data Fig. 7e,f). Following this general characterization, we measured CK-B mRNA/protein and phosphocreatine/creatine levels in WAT. We found that mice fed HFD exhibited lower levels of *Ckb* mRNA (Fig. 7a) and CK-B protein, where the latter was observed both in total tissue lysates and in intact tissue by immunofluorescence analysis (Fig. 7b,c). This was accompanied by higher phosphocreatine levels and phosphocreatine/creatine ratio compared to mice on chow diet (CD; Fig. 7d). In concordance with the findings in our clinical cohorts, these results confirm that the phosphocreatine/creatine pathway is perturbed by weight gain in mice. To determine if elevating phosphocreatine levels was sufficient to enhance WAT inflammation in vivo, we administered phosphocreatine (3 mg g^−1^) or phosphate buffered saline (PBS) intraperitoneally in chow-fed mice daily for seven days. This short-term treatment resulted in significant increases in WAT phosphocreatine levels (Fig. 7e) but did not influence body weight, WAT weight, fasting plasma glucose and insulin (Extended Data Fig. 7g–j) or *Ckb* mRNA and protein levels (Fig. 7f,g). However, we observed an increase in the expression of *Ccl2* and *Cd68* (with a trend for an increase in *Adgre1*) (Fig. 7f) and increased immunofluorescence staining of F4/80 following phosphocreatine injections (Fig. 7h).

**Fig. 8:**
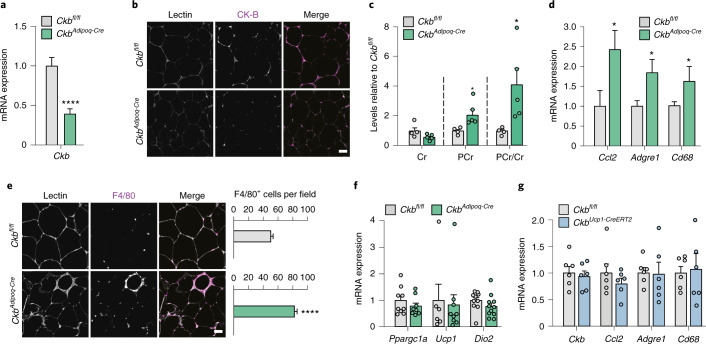
Adipocyte-specific *Ckb* deletion induces WAT phosphocreatine accumulation and inflammation in vivo. **a**, Male mice with an adipocyte-specific deletion of *Ckb* (*Ckb*^*Adipoq-Cre*^) and control littermates (*Ckb*^*fl/fl*^) were fed a HFD for 16 weeks starting at four weeks of age. Effects on body weight and glucose tolerance of this cohort have been presented^17^. *Ckb* gene expression was determined in pgWAT by qPCR (*n* = 11 mice per group). ****P < 0.0001. **b**, Representative immunofluorescence microphotographs of pgWAT from *Ckb*^*Adipoq-Cre*^ and *Ckb*^*fl/fl*^ mice. Sections were stained with *Lens culiniaris* agglutinin (Lectin) and antibodies targeting CK-B. Scale bar, 50 μm. **c**, Phosphocreatine and creatine levels in pgWAT from *Ckb*^*Adipoq-Cre*^ (*n* = 5) and *Ckb*^*fl/fl*^ (*n* = 4) mice. *P = 0.040 for phosphocreatine and 0.045 for phosphocreatine/Cr, respectively. **d**, mRNA levels for genes encoding inflammatory proteins in pgWAT from *Ckb*^*Adipoq-Cre*^ and *Ckb*^*fl/fl*^ mice (*n* = 11 per group). *P* = 0.018 for *Ccl2*, 0.04 for *Adgre1* and 0.05 for *Cd68*, respectively. **e**, Same as **b** but sections were stained with F4/80-targeting antibodies. The number of F4/80^+^ cells was counted in three to four random fields (*n* = 4 mice per group). Scale bar, 50 μm. ****P < 0.0001. **f**, mRNA levels for genes encoding thermogenic markers in pgWAT from *Ckb*^*Adipoq-Cre*^ and *Ckb*^*fl/fl*^ mice (*n* = 6-11 mice). **g**, Male mice with a brown adipocyte-specific deletion in *Ckb* expression (*Ckb*^*Ucp1-CreERT2*^) and control littermates (*Ckb*^*fl/fl*^) were fed a HFD for 16 weeks. Expression of *Ckb* and inflammatory genes was determined in pgWAT (*n* = 5-6 mice per group). Data were analysed by Student’s two-sided *t*-test. Data are shown as mean ± s.e.m.

As intraperitoneal injections with high doses of phosphocreatine may affect multiple organs, we determined the effects of *Ckb* depletion on WAT inflammation in vivo by comparing adipocyte-specific *Ckb* deleted (*Ckb*^*Adipoq-Cre*^) with control (*Ckb*^*fl/fl*^) mice fed a HFD for 16 weeks. These samples were obtained from a published study where adipocyte-specific *Ckb* depletion promoted obesity and glucose intolerance^17^. However, due to the higher food consumption of control mice at the end of the intervention, both groups of mice displayed similar body weight/composition although the *Ckb*^*Adipoq-Cre*^ mice were more glucose intolerant and insulin-resistant^17^. We confirmed that the *Ckb*^*Adipoq-Cre*^ animals expressed lower *Ckb* mRNA levels (Fig. 8a) and CK-B protein in WAT (Fig. 8b), which was accompanied by an increase in phosphocreatine levels and the phosphocreatine/creatine ratio without affecting creatine abundance (Fig. 8c). Transcriptional measurements showed that *Ccl2*, *Adgre1* and *Cd68* mRNA levels were higher in WAT of *Ckb*^*Adipoq-Cre*^ mice compared to controls (Fig. 8d). We further validated the pro-inflammatory effect by performing immunofluorescence staining of F4/80. Our results show that macrophage infiltration was increased in the WAT of *Ckb*-depleted animals versus control littermates (Fig. 8e). As the *Ckb*^*Adipoq-Cre*^ lack CK-B in both brown and WAT, and display a defect in brown adipose tissue thermogenesis^17^, we next examined if WAT inflammation was secondary to impaired adipocyte thermogenesis. However, the expression of the thermogenic adipocyte-markers *Ppargc1a*, *Ucp1* and *Dio2*, was unaltered in the WAT of *Ckb*^*Adipoq-Cre*^ mice compared to control mice (Fig. 8f). Finally, we tested if *Ckb* depletion specifically in brown adipocytes results in WAT inflammation. Compared to *Ckb*^*fl/fl*^ mice, *Ckb*^*Ucp1-CreERT2*^ HFD-fed mice did not show any alterations in *Ckb*, *Ccl2*, *Adgre1* and *Cd68* mRNA levels in WAT (Fig. 8g). Together, these data demonstrate that perturbed phosphocreatine/creatine metabolism in WAT is linked to inflammation in both mice and humans.

## Discussion

The phosphocreatine/creatine energy shuttling system is highly conserved across species. Despite the fact that this pathway was discovered more than 180 years ago^39^, its physiological role in organs other than the brain and skeletal/cardiac muscle remains poorly understood. Creatine metabolism modulates multiple aspects of brown adipocyte and immune cell biology^16^. More specifically, creatine promotes thermogenesis through stimulation of mitochondrial ATP turnover in brown adipose tissue^17,29^ and has been linked to M2-polarization in macrophages^18^. While a recent study suggested that white adipocytes in breast tissue can supply neighbouring tumour cells with creatine^20^, the role of this pathway in WAT is unclear. Herein, we demonstrate that white adipocyte phosphocreatine/creatine metabolism is perturbed in the obese state of both humans and mice. This results in altered ATP/ADP levels, which in turn attenuate AMPK activity leading to increased transcription of multiple pro-inflammatory genes including the chemokine *CCL2*. Collectively, our data unravel a link between energy shuttling and inflammation in white adipocytes and suggest that this balance is perturbed in obesity.

Creatine kinases constitute a family of enzymes interconverting phosphocreatine and creatine^16^. They are encoded by four distinct genes, whose products localize to mitochondria and/or cytosol and display different directionalities based on substrate availability, as well as cell type-specific expression patterns. Our systematic mapping of these enzymes shows that human white adipocytes predominatly express CK-B and CK-MT2, where the former localizes to the cytosol and the latter is present in mitochondria. As the reverse creatine kinase reaction is known to dominate in the cytosol, changes in CK-B activity alters the capacity to convert phosphocreatine to creatine. In human and murine obesity, the levels of CK-B in white adipocytes are selectively downregulated, which is linked to an increase in phosphocreatine levels and the phosphocreatine/creatine ratio. We further demonstrate that *CKB* depletion in human white adipocytes mirrors this alteration and leads to an increase in glycolysis, mitochondrial activity and an accumulation of ATP. Our interpretation of these results is that perturbations in the creatine shuttle are sensed by the cells and result in compensatory changes in other bioenergetic pathways. The mechanisms linking these processes are currently unclear.

Evidence from studies of immune cells suggests that aerobic glycolysis and mitochondrial activity are closely related to inflammation and cytokine secretion^30,40,41^. As a result, we hypothesized that this constituted a link between *CKB* depletion and the induction of a pro-inflammatory response. To test this model, we treated human white adipocytes and mice with phosphocreatine and observed a similar induction of several pro-inflammatory genes without changes in mitochondrial activity. Instead, the common denominator between *CKB* depletion and phophocreatine incubation was an increase in the intracellular ATP/ADP ratio and an attenuation of AMPK activity. Treatments with multiple different AMPK activators abrogated the effects on CCL2 production induced by *CKB* depletion/phosphocreatine incubation, suggesting that AMPK coordinates these pathways. In line with this, AMPK regulates inflammatory processes in adipose tissue^42,43^ and we provide evidence that reduced AMPK activity (e.g., via knockdown of *PRKAA1 and PRKAG1*) increases *CCL2* expression. In addition, AMPK activity is reduced in both obese rodents^44^ and humans^45,46^, and targeting this pathway has been proposed as a potential therapeutic avenue in metabolic disease^47,48^.

Previous studies have demonstrated cross-talks between phosphocreatine/creatine and AMPK in skeletal muscle^49^. More specifically, biochemical approaches revealed that an increased phosphocreatine/creatine ratio directly inhibits AMPK activity and that CK-M interacts physically with AMPK. Whether the latter pertains to CK-B is unclear, but our data revealed that AMPK activation abrogated the effects of *CKB* depletion on *CCL2* expression in white adipocytes. As a result, clinically, the beneficial effects of metformin in reversing insulin resistance may partly be mediated downstream of phosphocreatine accumulation in adipocytes. Metabolic comparisons of metformin and selective AMPK activation, as well as *CKB* induction in experimental in vivo models of obesity may address this relationship. Unfortunately, at present, these studies are limited by the lack of specific *CKB* regulators.

White adipocytes play a central role in regulating WAT inflammation by secreting a large number of pro-inflammatory cytokines and chemokines^1^. While a chronic release of these factors by adipocytes clearly results in immune cell infiltration and disturbed tissue function, a transient adipocyte pro-inflammatory state is essential for tissue remodelling and healthy expansion^50,51^. However, despite decades of research, the signals triggering the pro-inflammatory response of white adipocytes remain largely unclear. Our present data suggest that the phosphocreatine/creatine system regulates adipocyte inflammation by intracellular energy shuttling. We speculate that *CKB* downregulation in the obese state leads to phosphocreatine accumulation, perturbed intracellular energy metabolism, attenuated AMPK activity and the development of a chronic inflammatory response. The results presented herein and previously^17^ show that adipocyte-specific *Ckb* depletion results in obesity, WAT inflammation and insulin resistance. Importantly, the effects we report herein are not secondary to changes in brown adipose tissue thermogenesis or WAT beigeing as evidenced by expression analyses in *CKB*-depleted human adipocytes and WAT of *Ckb*^*Adipoq-Cre*^ and *Ckb*^*Ucp1-CreERT2*^ mice. Altogether, our work highlights the importance of intracellular metabolites in coordinating metabolic and inflammatory processes in white adipocytes.

The factors controlling adipocyte *CKB* transcription remain unknown. In other tissues, *CKB* expression is regulated by adrenergic signalling and/or mechanical/oxidative stress^17,52,53^. Whether such regulatory events operate in white adipocytes remains unclear, but it is worth noting that all of these processes (for example, adipocyte death, hypoxia, ER stress and mechanical stress) are altered in obesity. Identification and perturbation of *CKB* regulatory elements was beyond the scope of the present study. Instead, we used two different approaches, that is phosphocreatine treatment and genetic *CKB*/*Ckb* depletion models, to alter the balance of this system. Admittedly, the phosphocreatine concentrations used in cells/animals were higher than those observed in the circulation (reported to be in the μM range^54^), which warrants caution in interpreting the data from these experiments. However, as a proof-of-concept these experiments indicate that the accumulation of phosphocreatine in WAT is sufficient to trigger the expression of downstream inflammatory genes. Finally, all studies in humans were performed in subcutaneous WAT and we cannot establish whether the same mechanisms are relevant in other WAT regions.

Based on a translational study design encompassing clinical cohorts and human as well as murine in vitro/in vivo models, we provide evidence that the phosphocreatine/creatine pathway is a determinant of pro-inflammatory status in white adipocytes. Our findings suggest that intracellular energy homeostasis is coordinated with WAT inflammation. Future studies may reveal whether nutritional/pharmacological interventions targeting phosphocreatine/creatine metabolism can improve the metabolic consequences linked to obesity.

## Methods

### Materials

Materials/reagents are listed with catalogue numbers and vendors in Supplementary Table 2.

### Human subjects

Clinical data for all four cohorts are presented in Supplementary Table 1. All studies were approved by the regional ethics board and informed written consent was obtained from all participants.

### Animal studies

For HFD and phosphocreatine injection studies, pathogen-free C57BL/6J male mice were purchased from Charles River (Germany). Mice were housed at the KM-B animal facility at the Karolinska Institutet in ventilated cages (four animals per cage) with a 12 h light/12 h dark cycle (lights on 06:00–18:00) in a temperature-controlled (20–24 °C, 50% humidity) facility with ad libitum access to food and water. Animals were handled following the European Union laws and guidelines for animal care. Health inventories were performed on a regular basis (every 3 months) and followed the guidelines of the Federation of European Laboratory Animal Science Associations. All experimental procedures were approved by the Stockholm North Animal Ethical Committee (ethical permit N38/15), and special care was taken to minimize animal suffering and to reduce the number of animals used. For HFD experiments, five- to six week-old mice were fed either with CD (4% kcal from fat, R34; Lantmännen) or HFD (60% kcal from fat, D12492i, Research Diets) for five weeks. For phosphocreatine injection experiments, mice were fed a standard rodent CD (4% kcal from fat, R34; Lantmännen). At 11 weeks of age, mice with similar body weights were randomized (*n* = 8 per group) to receive daily intraperitoneal injection of phosphocreatine (3 mg g^−1^ body weight) or PBS (20 ml kg^−1^ body weight) for seven days. This time span was based on the regulations of the animal ethical committee. Four hours before the sacrifice, body weight was measured. Animals were euthanized under general anaesthesia by avertin injection and the wet weight of each dissected tissue was measured. WAT was obtained from the perigonadal (pgWAT) and inguinal (iWAT) depots which were weighed. Samples for qPCR, metabolite and protein analyses were snap-frozen in liquid nitrogen immediately after the wet weight was determined. One part of the fresh tissue samples was fixed in 4% formalin (pH 7.0) and used for immunofluorescence analyses as described below. The generation, housing and diet intervention of *Ckb*^AdipoQ-Cre^, *Ckb*^*Ucp1-CreERT2*^ and *Ckb*^*fl/fl*^ animals has been described^17^.

### Metabolic evaluations in mice

Glucose tolerance in CD-/HFD-fed animals was determined two days before sacrifice. In brief, glucose (1 g kg^−1^body weight) was administered by intraperitoneal injection in mice fasted for 4 h. Blood was sampled through the tail vein to measure glucose (OneTouch Ultra 2 glucose meter; LifeScan). Plasma insulin levels were measured by a commercial ELISA kit (Crystal Chem) according to the manufacturer’s instructions.

### Immunofluorescence analyses

WAT samples were fixed in 4% paraformaldehyde (PFA) for 24 h, embedded in paraffin and then sectioned (5 μm) and stained with hematoxylin and eosin (Sigma-Aldrich). Immunofluorescence was performed as described^55^. Slides were incubated overnight with an Alexa-488-coupled anti-F4/80 (1:50) or anti-CKB (1:100) antibody. Goat anti-Rabbit Rhodamine Red-X (1:500) was used a as a secondary antibody in the CKB experiments. Lens culinaris agglutinin (1:500) and Hoescht (1:500) were applied for 20 min to counterstain plasma membranes and nuclei, respectively. For each section, the total number of F4/80 positive cells were counted in three to four random ×10 fields using a Axio Observer.Z1 inverted fluorescence microscope (Zeiss) and the AxioVision software.

Adipocyte progenitors were stained for DPP4. In brief, cells were seeded on glass coverslips (thickness no. 1.5) and fixed in 4% PFA for 15 min at room temperature. Cells were washed twice with PBS, permeabilized using 0.1% Triton-X100 for 10 min and blocked for 30 min in PBS containing 10% normal goat serum. Subsequently, cells were incubated for 1 h with an antibody targeting DPP4 in blocking buffer. Cells were washed three times with PBS and incubated with secondary Alexa Fluor-conjugated antibodies for 1 h. Following three washes with PBS, cells were stained with Hoechst (diluted 1:5,000) for 15 min and mounted in fluorescence mounting medium (Fluoromount Aqueous Mounting Medium, refractive index 1.4). Images were acquired using a CREST V3 confocal system (Crest Optics) mounted on an inverted Nikon Ti2 microscope equipped with a Prime BSIexpress sCMOS camera (pixel size 6.5 μm) from Photometrics. A Nikon ×20/0.75 air objective was used to acquire images.

For TOM20 imaging in differentiated adipocytes, cells transfected with either si*C* or si*CKB* were seeded on no. 1.5 glass coverslips and fixed in 4% PFA for 15 min at room temperature. Staining was performed as described above for adipocyte progenitors, using an antibody targeting TOM20. Images were acquired using a CREST V3 confocal system (Crest Optics) in widefield mode (no spinning disk inserted) mounted on an inverted Nikon Ti2 microscope equipped with a Prime 95B sCMOS camera (pixel size 11 μm) from Photometrics. A Nikon ×60/1.4 oil objective was used to acquire images. The ×1.5 tube lens of the Ti2 was inserted to reach Nyquist sampling in *xy*. A *z*stack was acquired at Nyquist sampling in *z* and the stack was deconvolved using Nikon NIS element software and the fast deconvolution algorithm. Mitochondrial content in differentiated adipocytes was further evaluated by measurement of fluorescence intensity in in vitro adipocytes using MitoTracker Deep Red FM according to manufacturer’s instructions.

### Cell culture

Cultures of in vitro differentiated human adipocytes were established and differentiated as described^19^. In brief, cells were obtained from abdominal subcutaneous WAT of one male (16 years, BMI 24 kg m^−^^2^) and one female (39 years, BMI 23.6 kg m^−^^2^) donor.

### RNAi experiments and incubations with chemical compounds

Short interfering oligonucleotides (siRNAs) were introduced by electroporation using a Neon Transfection System (1,300 V, 20 ms, 2 pulses) 100 µl Kit (Invitrogen) in human in vitro differentiated adipocytes at day eight of differentiation. All transfections were performed using a final concentration of 20 nmol l^−1^ siRNA oligonucleotides and were compared with non-silencing control RNAi. In the indicated experiments, at day 13 post-adipogenic induction, human in vitro differentiated adipocytes were treated with phosphocreatine (7.5–30 mmol l^−1^), creatine (7.5–30 mmol l^−1^), oligomycin (1 μmol l^−1^), 2-deoxy-d-glucose (100 μmol l^−1^), Bongkrekic acid (1 μmol l^−1^), UK5099 (10 μmol l^−1^), ETO (3 μmol l^−1^), BPTES (10 μmol l^−1^), AICAR (250 μmol l^−1^), Metformin (10 mmol l^−1^), PF-739 (5 μmol l^−1^) and TNF-α (1.5 ng ml^−1^) for 24 h.

### Cellular triglyceride content and lipid staining

Cellular triglyceride levels were measured on cells plated in 96-well plates, using the Triglyceride Quantification Colorimetric/Fluorometric Kit (Sigma-Aldrich) according to the manufacturer’s instructions.

For imaging, cells transfected with either si*C* or si*CKB* were fixed in 4% PFA for 15 min at room temperature and washed twice with PBS. Cells were stained with BODIPY 493/503 (diluted 1:2,500, ThermoFisher) to stain accumulated lipids and Hoechst (diluted 1:5,000) for 15 min. Cells were then washed four times with PBS and images were acquired using CREST V3 confocal system (Crest Optics) mounted on an inverted Nikon Ti2 microscope equipped with a Prime BSIexpress sCMOS camera (pixel size 6.5 μm) from Photometrics. A Nikon ×60/1.4 oil objective was used to acquire images.

### Glucose uptake assay

Glucose uptake was determined by measuring radioactivity within cells by liquid scintillation counting. In brief, the day before the assay, insulin was removed from the culture medium. After two washes with PBS, cells were incubated for 50 min at 37 °C in insulin-free DMEM (Biochrom AG). Then, 125 μmol l^−1^ 2-deoxy-d-glucose and 0.4 μCi 2-deoxy-[1-^3^H]-d-glucose per well were added for 10 min. Subsequently, the cells were washed three times with cold PBS and lysed in 0.1% SDS/H_2_O. One part of the lysate was saved for determination of protein concentrations using Pierce BCA Protein determination kit (ThermoFisher). The rest of the lysate was transferred to cuvettes containing scintillation fluid and counts per minute was recorded using a Liquid Scintillation Analyser (Tri-Carb 4910 TR, PerkinElmer) and Optiphase Hisafe 3 (PerkinElmer) as scintillation fluid. Data were normalized to protein concentration.

### Seahorse assays and CyQUANT analysis

OCRs were measured with an XF96 Seahorse Extracellular Flux Analyzer (Agilent) using Cell Mito Stress Test kits. For measurements of basal OCR, cells were incubated in medium supplemented with 1 mmol l^−1^ pyruvate, 2 mmol l^−1^ glutamine and 10 mmol l^−1^ glucose. To assess maximal respiration, FCCP (uncoupling agent) was titrated from 1–2 μmol l^−1^. Then, Mito Stress assays were performed by sequential addition of 1.5 μmol l^−1^ oligomycin (inhibitor of ATP synthesis), 1.5 μmol l^−1^ FCCP and 0.5 μmol l^−1^ rotenone/antimycin A (inhibitors of complex I and complex III of the respiratory chain, respectively). Mitochondrial ATP production was determined by subtracting the oligomycin-depressed OCR from basal OCR. Mitochondrial fuel stress tests were performed using the XF96 fuel kit after acute injection of UK5099 or BPTES/ETO. Seahorse data were normalized using ThermoFisher Scientific’s fluorescent CyQUANT Kit (ThermoFisher) according to manufacturer’s instructions. Immediately after Seahorse analysis, the cells were incubated with the CyQUANT reagent and fluorescence was measured. For estimation of the basal and maximal respiration, the mean non-mitochondrial respiration was subtracted from the mean values of basal and maximal respiration.

### Determination of CCL2 and adiponectin secretion and plasma high-sensitivity C-reactive protein

For analyses of condtioned media of in vitro differentiated human adipocytes, samples were collected at day 14 of differentiation and secretion of CCL2 as well as adiponectin was determined by ELISA according to the manufacturer’s instructions.

### ATP/ADP analyses

Measures of cellular ATP and ADP levels were performed in cell lysates using the ADP/ATP Ratio Assay Kit (Sigma-Aldrich) according to the manufacturer’s instructions.

### Creatine kinase activity assay

Creatine kinase activity was determined with the Creatine Kinase Assay Kit (Abnova) in tissue/cell lysates according to the manufacturer’s specifications.

### RNA isolation, complementary DNA synthesis and real-time qPCR

Total RNA was extracted from intact human or murine WAT and cell cultures as described^19^. The concentration, purity and quality of the RNA were measured using Nanodrop 2000 (ThermoFisher) and Agilent 2100 Bioanalyzer (Agilent). Total RNA was reverse transcribed with iScript complementary DNA synthesis kits (BioRad). Assessments of mRNA levels were performed using TaqMan (Applied Biosystems) or SYBR-green (BioRad) assays and relative expression was calculated with the comparative Ct-method, that is, 2^ΔCt-target gene^/2^ΔCt-reference gene^. All primers and kits, including house-keeping genes, are listed in Supplementary Table 2.

### Transcriptomic array

Biotinylated DNA targets were prepared from 150 ng total RNA using the GeneChip WT Plus Reagent Kit according to the manufacturer’s instructions. Hybridization, washing and staining was carried out on Clariom S Human arrays, using Affymetrix GeneChip Fluidics Station 450 according to the manufacturer’s protocol. The fluorescent intensities were determined with Affymetrix GeneChip Scanner 3000 7G. Data were analysed with packages available from Bioconductor (http://www.bioconductor.org). Normalization and calculation of gene expression was performed with the Robust Multichip Average expression measure using the oligo package^56^. A non-specific filter was first applied to include genes with log_2_ expression signal >5 in at least 50% of all samples. Principal component analysis was then performed using the FactomineR package and Limma package^57^ was used to identify the differentially expressed genes that were ranked using *t-*statistics in the gene set enrichment analyses (GSEA)^58^. To identify genes/pathways affected by altered CK-B mRNA/protein levels in human adipocytes, GSEA was performed using clusterProfiler package^59^ with Hallmark gene sets from MsigDB (http://www.gsea-msigdb.org/gsea/msigdb/index.jsp). GSEA of genes correlating with *CKB* expression in human WAT was performed using the available transcriptomics data from cohort 2 (ref. ^21^). In brief, probesets were merged at the gene level using collapseRows function (default setting) in WGCNA package^60^. Genes were then ranked based on their association with *CKB* using Spearman’s rank correlation coefficient (*ρ*) before performing GSEA.

### Western blot analyses

Western blots were performed as described previously^19^. All antibodies are listed in Supplementary Table 2. For blots where the proteins of interest run at the same size, lysates were subdivided in equal amounts and loaded on two separate gels.

### Isolation of mitochondria and cytoplasm from in vitro differentiated human adipocytes

in vitro differentiated human adipocytes were homogenized in ice-cold isolation buffer (250 mmol l^−1^ sucrose buffer, 20 mmol l^−1^ Tris-HCl (pH 7.4), 1 mmol l^−1^ EDTA (pH 8), 2% BSA) using a 27G syringe. The lysate was centrifuged at 1,000*g* for 15 min to remove the nuclei. The supernatant was centrifuged at 8,500*g* for 15 min to isolate mitochondria and the mitochondrial pellet was resuspended in TES buffer (100 mmol l^−1^ KCl, 20 mmol l^−1^ TES, 1 mmol l^−1^ EGTA, 0.6% BSA (pH 7.2)). After mitochondrial isolation, the supernatant was centrifuged for 2 h at 20,000*g* to remove the membranes and separate the lipid droplets. The remaining supernatant was used as cytoplasmic fraction.

### Targeted metabolite analyses in cells and murine tissues

Metabolites were profiled at the Swedish Metabolomics Centre, the Helmholtz Zentrum and at McGill University. For the latter, the analyses were performed on WAT from *Ckb*^*Adipoq-Cre*^ and *Ckb*^*fl/fl*^ and the methods have been described^17^. For analyses performed at the Swedish Metabolomics Centre, human in vitro differentiated adipocytes were lysed at day 14 post-induction of differentiation in 1ml of 90% methanol (diluted H_2_O) containing 0.5 µmol l^−1^ creatine-D_3_ as an internal standard, shaken with metal beads at 30 kHz for 3 min and centrifuged at 14,000*g* for 10 min. The supernatants were divided in two aliquots for both liquid (LC) and gas (GC) chromatography analyses. Phosphocreatine and creatine were analysed using LC–tandem mass spectrometry. The separation was achieved by using hydrophilic interaction chromatography (iHILIC-(P) Classic, PEEK, 150 mm × 2.1 mm, 5 µm, HILICON). The LC–tandem mass spectrometry system consisted of an Agilent 1290 UPLC connected to an Agilent 6490 triple quadrupole tandem mass spectrometer (Agilent). Analytes were ionized in electrospray source operated in the positive (creatine) and negative (phosphocreatine) mode. The analyses were performed in multiple reaction monitoring mode. Glycolytic intermediates were analysed by GC–MS. Before the evaporation of the supernatant, it was spiked with 1.05 ng of each GC–MS internal standards. Derivatization was performed as described^61^. The GC–MS system consisted of an Agilent 7693 autosampler, an Agilent 7890A gas chromatograph and an Agilent 7000C QQQ mass spectrometer. Ions were generated by a 70 eV electron beam and analysed in dynamic multiple reaction monitoring mode. Data were processed and analysed using MassHunter Qualitative Analysis, Quantitative Analysis (QqQ; Agilent) and Excel (Microsoft) software. For analyses performed at the Helmoltz Zentrum Munchen, metabolites were extracted from cell pellets of in vitro differentiated adipocytes using 1 ml of cold CHCl_3_/MeOH/H_2_O (1/3/1, v/v/v). After suspension in solvent, cells were transferred to 2 ml MN Bead Tubes Type A (Macherey-Nagel) and lysed using a Precellys Bead Beating system with an additional Cryolys cooling module (Bertin Instruments). After lysis, samples were incubated for 10 min in an ice-cold ultrasonic bath followed by centrifugation at 4 °C for 15 min. The supernatant was transferred to a fresh reaction tube and evaporated to dryness using a centrifugal evaporator. Proteins were extracted from the residue cell debris pellet and quantified using a BCA Kit (Sigma-Aldrich). Metabolite profiling was performed using a Sciex Exion AD LC coupled to a Sciex X500R Q-ToF-MS (Sciex). Separation was achieved on an Agilent InfinityLab Poroshell 120 HILIC-Z column (2.1 mm × 150 mm, 2.7 µm particle size, PEEK-lined) (Agilent Technologies). Analysis conditions were similar to Hsiao et al.^62^. Dried samples were re-dissolved in 50 µl CHCl_3_/MeOH/H_2_O (1/3/1, v/v/v) and 40 µl were transferred to an autosampler vial and 10 µl to a pooled quality control sample. These samples were used for conditioning of the column and were also injected every ten samples. Data analysis was performed in Sciex OS (Sciex). Peaks for creatine (C_4_H_9_N_3_O_2_, [M + H]^+^, 10.42 min) and phosphocreatine (C_4_H_10_N_3_O_5_P, [M + H]^+^, 13.63 min) were integrated with an extracted ion chromatogram (width of 0.02 Da and a Gaussian smooth width of three points using the MQ4 peak picking algorithm. Identity of peaks was confirmed using authentic standards of all substances and comparison against reference spectra and peak areas were normalized according to the protein content of the respective sample.

### Statistics

Data are reported as mean ± s.e.m. unless otherwise stated. Results were compared by one- or two-tailed Student’s *t*-test or one- or two-way analysis of variance (ANOVA) with Tukey´s post-hoc test. For correlation analyses in clinical cohorts, simple and multiple regression analyses (with BMI included as independent regressor) were used. The number of independent experiments and relevant statistical methods for each panel are detailed in the figure legends. Statistical analyses of cell and animal data were performed using Prism (GraphPad Software), analyses of clinical data were done using JMP (v.15.1, SAS) and bioinformatic analyses were performed with R v.4.1.1.

### Reporting Summary

Further information on research design is available in the Nature Research Reporting Summary linked to this article.

## Supplementary information

Supplementary Information Supplementary Tables 1 and 2.

Reporting Summary

## Data Availability

Microarray data generated or retrospectively analysed in this study are publicly available in the NCBI Gene Expression Omnibus repository under the accession numbers GSE25401 (transcriptomics of cohort 2), GSE59034 (transcriptomics of cohort 3), GSE100795 (transcriptomics of different resident cells of human WAT) and GSE192361 (transcriptomics of si*CKB-* versus si*C-*transfected cells). Metabolomics data from cohort 1 are provided in ref. ^19^. RNA sequencing data at multiple time points during adipocyte differentiation are available from these cells within the FANTOM5 project^63^ and was used to extract information on genes within the phosphocreatine/creatine pathway presented in Fig. 2b. Source data are provided with this paper. Additional data that support the findings of this study are available from the corresponding authors upon reasonable request.

## Source data

Source Data Fig. 1 Unprocessed western blots for Fig. 1.
Source Data Fig. 2 Unprocessed western blots for Fig. 2.
Source Data Fig. 6 Unprocessed western blots for Fig. 6.
Source Data Fig. 7 Unprocessed western blots for Fig. 7.
Source Data Extended Data Fig. 3 Unprocessed western blots for Extended Data Fig. 3.
Source Data Extended Data Fig. 6 Unprocessed western blots for Extended Data Fig. 6.
