# Supplementary material for: Impaired phosphocreatine metabolism in white adipocytes promotes inflammation
Source: Nat Metab. 2022 Feb 14;4(2):190–202. doi: 10.1038/s42255-022-00525-9 (PMC8885409; doi:10.1038/s42255-022-00525-9)
Supplement: Supplementary file 1 — Supplementary Tables 1 and 2. [file 42255_2022_525_MOESM1_ESM.pdf]

nature portfolio

https://doi.org/10.1038/s42255-022-00525-9

Supplementary information

Impaired phosphocreatine metabolism in white adipocytes promotes inflammation

In the format provided by the authors and unedited

Cohort 1 (NCT01727245)			
**Parameters**	**Non-obese (n=13)**	**Obese (n=13)**	**p-value**
Age (years)	48 (26-60)	45 (35-53)	0.89
BMI (kg/m^2)	22.5 (20.5-25.2)	37.9 (36.9-39.2)	<0.0001
Body fat (%)	28.7 (23.5-31.9)	50.8 (49.4-53.4)	<0.0001
Fat cell volume (pL)	370 (239-505)	837 (674-1145)	<0.0001
P-glucose (mmol/L)	5 (4.8-5.8)	5.4 (4.9-5.8)	0.2
P-insulin (mU/L)	5.54 (3.33-6.51)	13 (6.85-19.9)	<0.0001
P-cholesterol (mmol/L)	4.8 (4.1-5)	5 (4.1-5.7)	0.26
P-triglycerides (mmol/L)	0.74 (0.63-1.1)	1.5 (1-2)	0.0002
P-FFA (mmol/L)	0.63 (0.45-0.71)	0.74 (0.56-1.18)	0.07
P-HDL (mmol/L)	1.8 (1.4-2)	1.2 (0.9-1.5)	0.0001

Cohort 2			
**Parameters**	**Non-obese (n=26)**	**Obese (n=30)**	**p-value**
Age (years)	41.5 (35-48)	41.5 (38-45)	0.83
BMI (kg/m^2)	24.1 (23-25.1)	41.35 (36.9-43.4)	<0.0001
Body fat (%)	30.63 (27.63-32.38)	55.66 (47.88-59.06)	<0.0001
Fat cell volume (pL)	472.5 (370-580)	925.5 (833-996)	<0.0001
P-glucose (mmol/L)	4.75 (4.6-5)	5.25 (5-5.5)	0.01
P-insulin (mU/L)	4.35 (3.3-5.9)	14.6 (8.9-18.3)	<0.0001
P-triglycerides (mmol/L)	0.78 (0.7-0.99)	1.45 (1.1-1.8)	0.0006
P-FFA (mmol/L)	0.66 (0.56-0.78)	0.77 (0.7-0.89)	0.045

Cohort 3 (NCT01785134)				
**Parameters**	**Obese (n=15)**	**Post-surgery (n=15)**	**Control (n=15)**	**p-value**
Age (years)	50 (41-53)	52 (43-54)	48 (42-54)	0.82
BMI (kg/m^2)	40 (37.6-43.4)	25.5 (22.6-27.5)	25.3 (23.5-28.0)	<0.0001
Body fat (%)	49.7 (47.5-53.2)	31 (27.0-34.7)	33.6 (26.6-40.4)	<0.0001
Fat cell volume (pL)	976 (769-1101)	322 (210-460)	504 (382-645)	<0.0001
P-glucose (mmol/L)	5.4 (5.2-5.9)	4.8 (4.4-5.0)	5.2 (5.0-5.4)	0.0004
P-insulin (mU/L)	12.5 (8.9-14.2)	3.35 (2.5-5.6)	3.85 (3.0-5.7)	<0.0001
P-cholesterol (mmol/L)	5 (4.3-5.8)	4 (3.7-4.3)	4.6 (4.5-6.0)	0.023
P-triglycerides (mmol/L)	1.5 (0.92-2.8)	0.99 (0.61-1.1)	0.7 (0.56-1.2)	0.0049
P-FFA (mmol/L)	0.86 (0.69-0.95)	0.67 (0.40-1.2)	0.57 (0.48-0.63)	0.078
P-HDL (mmol/L)	1.2 (1-1.3)	1.5 (1.3-1.9)	1.55 (1.4-2)	0.0035

Cohort 4 (NCT01727245)			
**Parameters**	**Non-obese (n=17)**	**Obese (n=18)**	**p-value**
Age (years)	53 (44-65)	50 (39-56)	0.18
BMI (kg/m^2)	25.85 (24.30-27.30)	38.10 (36.40-39.90)	<0.0001
Body fat (%)	28.60 (26.40-32.90)	43.15 (39.80-46.40)	<0.0001
Fat cell volume (pL)	558 (469-629)	952 (717-1200)	0.003
P-glucose (mmol/L)	5.6 (5.4-5.8)	5.8 (5.4-5.9)	0.19
P-insulin (mU/L)	6.03 (3.74-7.99)	19.60 (12.2-38.9)	0.001
P-cholesterol (mmol/L)	4.9 (4.2-5.6)	5 (4.1-5.6)	0.91
P-triglycerides (mmol/L)	0.78 (0.64-1.5)	1.75 (1.1-2.2)	0.001
P-FFA (mmol/L)	0.46 (0.35-0.58)	0.57 (0.42-0.65)	0.19
P-HDL (mmol/L)	1.2 (0.9-1.5)	1 (0.8-1.2)	0.06

Supplementary Table 1: Clinical and biological parameters for subjects included in the four cohorts of the study. Data are expressed in median (95% CI). NCT numbers in clinicaltrials.gov are provided. P-values were determined using Student's t-test for cohort 1, 2 and 4 and one-way ANOVA for cohort 3.

Supplementary Table 2. List of reagents.

Reagents and Antibodies	SOURCE	IDENTIFIER
Lamin A/C	Cell Signaling Tech.	Cat#4777
Tubulin	Cell Signaling Tech.	Cat#2144
GAPDH (14C10)	Cell Signaling Tech.	Cat#2118
TOM20 (for western blot)	Cell Signaling Tech.	Cat#42406
TOM20 (for immunofluorescence)	Proteintech	Cat#66777-1-Ig
AMPKα	Cell Signaling Tech.	Cat#2532
Phospho-AMPKα	Cell Signaling Tech.	Cat#2535
Creatine kinase B (for immunofluorescence)	Abcam	Cat#AB151579
Creatine kinase B (for western blot)	Abcam	Cat#AB108388
CKMT2	Abcam	Cat#AB55963
Total OXPHOS	Abcam	Cat#AB110413
F4/80-Aexa Fluor 488	Abcam	Cat#AB204266-100
Goat α-Rabbit IgG (H+L)	Thermo Fisher	Cat#R-6394
LCA, Rhodamine	Vector lab	Cat#RL-1042
Rabbit IgG-HRP	Sigma-Aldrich	Cat#A9169
Mouse IgG-HRP	Sigma-Aldrich	Cat#5278
DPP4/CD26	Fisher Scientific	Cat#16847663
Actin	Sigma-Aldrich	Cat#A2066
Goat serum	Sigma-Aldrich	Cat#G9023

Critical Commercial Assays		
Seahorse XF Glycolysis Stress Test	Agilent	Cat#103020-100
Seahorse XF Mito Stress Test	Agilent	Cat#103015-100
Seahorse XFp Mito Fuel Flex Test	Agilent	Cat#103070-100
iScript cDNA Synthesis kit	Bio-Rad	Cat#170-8891
Insulin ELISA Kit	Crystal Chem	Cat#90080
Pierce BCA Protein Assay Kit	ThermoFisher	Cat#23227
MCP-1/CCL2 Human ELISA Kit	ThermoFisher	Cat#88-7399-22
Human Total Adiponectin/Acrp30 Quantikine ELISA	R & D systems	Cat#DRP300
Triglyceride quantification assay-Colorimetric/fluorometric kit	Sigma-Aldrich	Cat# MAK266-1KT
ATP/ADP ratio assay	Sigma-Aldrich	Cat#MAK135-1KT
Creatine kinase activity assay	Abnova	Cat#KA1665
MitoTracker™ Deep Red FM	ThermoFisher	Cat#M22426

Gene silencers		
ON-TARGETplus Non-targeting Pool	Dharmacon	Cat# D-001810-01-20
ON-TARGETplus siRNA CKB - Human	Dharmacon	Cat#L-006706-00-0005
ON-TARGETplus siRNA SLC6A8 - Human	Dharmacon	Cat#L-007608-00-0005
ON-TARGETplus siRNA CKMT2 - Human	Dharmacon	Cat#L-006709-00-0005
ON-TARGETplus siRNA PRKAA1 - Human	Dharmacon	Cat#L-005027-00-0005
ON-TARGETplus siRNA PRKAG1 - Human	Dharmacon	Cat#L-009056-00-0005
AllStars Negative Control siRNA (5 nmol)	Qiagen	Cat#1027280
Hs_CKB_7 FlexiTube siRNA	Qiagen	Cat#1027417

**Chemicals, Peptides, and Recombinant Proteins**		
TaqMan Master Mix	Applied Biosystems	Cat#4318157
SYBR-green Master Mix	Bio-Rad	Cat#1708884
Amersham ECL Prime Blocking Reagent	GE Healthcare	Cat#RPN418
Amersham ECL Prime Western Blotting Detection Reagent	GE Healthcare	Cat#RPN2232
DTT	Sigma-Aldrich	Cat#10197777001
Optiphase Hisafe 3	Perkin Eimer	Cat#1200.437
QIAzol lysis reagent	Qiagen	Cat#79306
Bodipy 493/503	ThermoFisher	Cat# D3922
Hoechst	ThermoFisher	Cat#34580
CyQUANT	ThermoFisher	Cat#C7026
2-deoxy-D- [1-H^3]-glucose	Perkin Eimer	Cat#NET328250UC
Glucose Solution	ThermoFisher	Cat#A2494001
RIPA buffer	ThermoFisher	Cat#89901
Collagenase	Sigma-Aldrich	Cat#C6885
2-deoxy-D-glucose	Sigma-Aldrich	Cat#D6134-1G
FGF2 human	Sigma-Aldrich	Cat#F0291
Insulin (liquid, ready to use, 10mg/ml, 5ml)	Sigma-Aldrich	Cat#I9278
T3	Sigma-Aldrich	Cat#T6397
Transferrin	Sigma-Aldrich	Cat#T8158
IBMX (3-Isobutyl-1-methylxanthin)	Sigma-Aldrich	Cat#I5879
Rosiglitazone (100 mg)	Cayman Chemicals	Cat#71740
Dexamethasone	Sigma-Aldrich	Cat#D1756
0.5% Trypsin/EDTA (10X)	Invitrogen (GIBCO)	Cat#15400-054
Penicilline G 10000U/ml/Streptomycine 10000µg/ml	Invitrogen (GIBCO)	Cat#15140-122
Hepes 1M	Invitrogen (GIBCO)	Cat#15630-056
Ham's F-12 Nutrient Mix	Invitrogen (GIBCO)	Cat#21765-037
DMEM, low glucose, pyruvate (+ glutamine)	Invitrogen (GIBCO)	Cat#31885-023
Pierce™ 16% Formaldehyde (w/v), Methanol-free	ThermoFisher	Cat#28906
4x Laemmli Sample Buffer	BioRad	Cat#1610747
PF-739	Aobious	Cat#AOB33584
Creatine anhydrous	Sigma-Aldrich	Cat#C0780-50G
Creatine phosphate	Millipore Sigma	Cat#10621714001
UK-5099	Sigma-Aldrich	Cat#PZ0160-5MG
Etomoxir sodium salt hydrate	Sigma-Aldrich	Cat#E1905-5MG
BPTES	Sigma-Aldrich	Cat#SML0601-5MG
Bongkrekic acid	Cayman Chemicals	Cat#19079
Oligomycin	Agilent	Cat#103015-100
AICAR	Sigma-Aldrich	Cat#A9978-25MG
Metformin	Sigma-Aldrich	Cat# PHR1084
Seahorse Glutamine solution	Agilent	Cat#103579-100
Seahorse glucose solution	Agilent	Cat#103577-100
Seahorse pyruvate solution	Agilent	Cat#103578-100

Oligonucleotides		
TaqMan probe human *LRP10*	Applied Biosystems	Hs00204094_m1
TaqMan probe human *PPIA*	Applied Biosystems	Hs04194521_s1
TaqMan probe human *CCL2*	Applied Biosystems	Hs00234140_m1
TaqMan probe human *PLIN1*	Applied Biosystems	Hs00193510_m1
TaqMan probe human *PPARG*	Applied Biosystems	Hs01115513_m1

TaqMan probe human *FABP4*	Applied Biosystems	Hs01086177_m1
TaqMan probe human *CKB*	Applied Biosystems	Hs00176484_m1
TaqMan probe human *SLC6A8*	Applied Biosystems	Hs00940515_m1
TaqMan probe human *PRKAA1*	Applied Biosystems	Hs01562308_m1
TaqMan probe human *PRKAG1*	Applied Biosystems	Hs01091629_g1
TaqMan probe human *UCP1*	Applied Biosystems	Hs00222453_m1
TaqMan probe human *PPARGC1A*	Applied Biosystems	Hs00173304_m1
TaqMan probe murine *Ccl2*	Applied Biosystems	Mm00441242_m1
TaqMan probe murine *ActB*	Applied Biosystems	Mm00607939_s1
TaqMan probe murine *Gapdh*	Applied Biosystems	Mm99999915_g1
TaqMan probe murine *Ckmt1*	Applied Biosystems	Mm00438221_m1
TaqMan probe murine *Ckmt2*	Applied Biosystems	Mm01285553_m1
SYBR green probe murine *Adgre1*	Sigma-Aldrich	Fw:TGACTCACCTTGTGGTCCTAA; Rv:CTTCCCAGAATCCAGTCTTTCC
SYBR green probe murine *Cd68*	Sigma-Aldrich	Fw:TGTCTGATCTTGCTAGGACCG; Rv:GAGAGTAACGGCCTTTTTGTGA
SYBR green probe murine *Ckb*	Sigma-Aldrich	Fw:AAGTTCTCGGAGGTGCTCAA Rv:CCGTTGCTCCATCTCAATG
