# Supplementary material for: Impaired phosphocreatine metabolism in white adipocytes promotes inflammation
Source: Nat Metab. 2022 Feb 14;4(2):190–202. doi: 10.1038/s42255-022-00525-9 (PMC8885409; doi:10.1038/s42255-022-00525-9)
Supplement: Supplementary file 2 — Reporting Summary [file 42255_2022_525_MOESM2_ESM.pdf]

nature portfolio

Corresponding author(s): Mejhert and Rydén NATMETAB-A21044659B

Last updated by author(s): Dec 23, 2021

# Reporting Summary

Nature Portfolio wishes to improve the reproducibility of the work that we publish. This form provides structure for consistency and transparency in reporting. For further information on Nature Portfolio policies, see our Editorial Policies and the Editorial Policy Checklist.

## Statistics

For all statistical analyses, confirm that the following items are present in the figure legend, table legend, main text, or Methods section.

| n/a | Confirmed | |
|---|---|---|
| ☐ | ☒ | The exact sample size ($n$) for each experimental group/condition, given as a discrete number and unit of measurement |
| ☐ | ☒ | A statement on whether measurements were taken from distinct samples or whether the same sample was measured repeatedly |
| ☐ | ☒ | The statistical test(s) used AND whether they are one- or two-sided<br>*Only common tests should be described solely by name; describe more complex techniques in the Methods section.* |
| ☐ | ☒ | A description of all covariates tested |
| ☐ | ☒ | A description of any assumptions or corrections, such as tests of normality and adjustment for multiple comparisons |
| ☐ | ☒ | A full description of the statistical parameters including central tendency (e.g. means) or other basic estimates (e.g. regression coefficient) AND variation (e.g. standard deviation) or associated estimates of uncertainty (e.g. confidence intervals) |
| ☐ | ☒ | For null hypothesis testing, the test statistic (e.g. $F$, $t$, $r$) with confidence intervals, effect sizes, degrees of freedom and $P$ value noted<br>*Give P values as exact values whenever suitable.* |
| ☒ | ☐ | For Bayesian analysis, information on the choice of priors and Markov chain Monte Carlo settings |
| ☒ | ☐ | For hierarchical and complex designs, identification of the appropriate level for tests and full reporting of outcomes |
| ☒ | ☐ | Estimates of effect sizes (e.g. Cohen's $d$, Pearson's $r$), indicating how they were calculated |

*Our web collection on statistics for biologists contains articles on many of the points above.*

## Software and code

Policy information about availability of computer code

| | |
|---|---|
| Data collection | AxioVision 4.9.1, NIS-Elements. |
| Data analysis | MassHunter, Excel 365, GraphPad Prism 7, JMP v15.1, SPSS v24, ImageJ 1.45 and R v4.1.1 |

For manuscripts utilizing custom algorithms or software that are central to the research but not yet described in published literature, software must be made available to editors and reviewers. We strongly encourage code deposition in a community repository (e.g. GitHub). See the Nature Portfolio guidelines for submitting code & software for further information.

## Data

Policy information about availability of data

All manuscripts must include a data availability statement. This statement should provide the following information, where applicable:

- Accession codes, unique identifiers, or web links for publicly available datasets
- A description of any restrictions on data availability
- For clinical datasets or third party data, please ensure that the statement adheres to our policy

Microarray data generated in this study are publicly available in the NCBI Gene Expression Omnibus repository under the accession number GSE192361. Additional data used in this work are available using GEO numbers detailed in the paper under the section Data Availability.

# Field-specific reporting

Please select the one below that is the best fit for your research. If you are not sure, read the appropriate sections before making your selection.

☒ Life sciences ☐ Behavioural & social sciences ☐ Ecological, evolutionary & environmental sciences

For a reference copy of the document with all sections, see nature.com/documents/nr-reporting-summary-flat.pdf

# Life sciences study design

All studies must disclose on these points even when the disclosure is negative.

| | |
|---|---|
| Sample size | Human omics data were retrospectively analyzed from previously described independent patient cohorts (see references in the Results section). For animal experiments, the number of mice was based on previous experience. No sample size calculation was therefore performed. |
| Data exclusions | No data were excluded |
| Replication | Experimental findings were repeated multiple times in independent experiments as indicated in the figure legends |
| Randomization | Experiments in wild-type and knockout animals were not randomized as mice were identified by genotyping. In other experiments, samples/mice treated with siRNA/drugs/diets, were randomly assigned to each group. |
| Blinding | For the mouse studies, the investigators were not blinded as they handled the phenotyping and sacrification of the mice. However, the investigators were blinded for sample processing and samples were processed in random order. |

# Reporting for specific materials, systems and methods

We require information from authors about some types of materials, experimental systems and methods used in many studies. Here, indicate whether each material, system or method listed is relevant to your study. If you are not sure if a list item applies to your research, read the appropriate section before selecting a response.

## Materials & experimental systems

| n/a | Involved in the study | |
|---|---|---|
| ☐ | ☒ | Antibodies |
| ☒ | ☐ | Eukaryotic cell lines |
| ☒ | ☐ | Palaeontology and archaeology |
| ☐ | ☒ | Animals and other organisms |
| ☐ | ☒ | Human research participants |
| ☐ | ☒ | Clinical data |
| ☒ | ☐ | Dual use research of concern |

## Methods

| n/a | Involved in the study | |
|---|---|---|
| ☒ | ☐ | ChIP-seq |
| ☒ | ☐ | Flow cytometry |
| ☒ | ☐ | MRI-based neuroimaging |

## Antibodies

| | |
|---|---|
| Antibodies used | All antibodies used in this study are detailed in Supplementary Table 2 including catalogue numbers and manufacturers. |
| Validation | All antibodies used in this study were commercially available and validation data can be found on the respective manufacturers' websites. Key findings were validated by siRNA knockdown experiments. |

## Animals and other organisms

Policy information about studies involving animals; ARRIVE guidelines recommended for reporting animal research

| | |
|---|---|
| Laboratory animals | For Ckb floxed animals, age and housing conditions are detailed in Rahbani et al (PMID 33597756).<br>For HFD and phosphocreatine injection studies, C57BL/6J male mice were used and were sacrificed at 10-12 weeks of age. Animals were housed at the KM-B animal facility at the Karolinska Institutet in ventilated cages (4 animals/cage) with a 12 hour light/12 hour dark cycle (lights on 06:00–18:00) in a temperature-controlled (20-24°C, 50 % humidity) facility with ad libitum access to food and water. Animals were handled following the European Union laws and guidelines for animal care. Health inventories were performed on a regular basis (every 3 months) and follow the guidelines of the Federation of European Laboratory Animal Science Associations. |
| Wild animals | No wild animals were included in the study. |
| Field-collected samples | No field-collected samples were used in this study. |

| | |
|---|---|
| Ethics oversight | Ethical permits were obtained from the regional ethics board as detailed in the manuscript. |

Note that full information on the approval of the study protocol must also be provided in the manuscript.

## Human research participants

Policy information about studies involving human research participants

| | |
|---|---|
| Population characteristics | Clinical characteristics of the four cohorts are detailed in Supplementary Table 1. |
| Recruitment | No new patients/research subjects were recruited for this study. |
| Ethics oversight | All studies based on existing human data sets were approved by the regional ethics board of the Stockholm County Council. Informed written consent was obtained from all participants. A compensation of approximately 100 EUR was given to participants undergoing fine needle biopsies according to Swedish guidelines and the approved ethical permits. |

Note that full information on the approval of the study protocol must also be provided in the manuscript.

## Clinical data

Policy information about clinical studies

All manuscripts should comply with the ICMJE guidelines for publication of clinical research and a completed CONSORT checklist must be included with all submissions.

| | |
|---|---|
| Clinical trial registration | NCT01727245 (cohort 1 and 4) and NCT01785134 (cohort 3) |
| Study protocol | The intervention (bariatric surgery) is described at clinicaltrials.gov |
| Data collection | All subjects were investigated in the morning after an overnight fast at the Karolinska University Hospital, Huddinge. Subcutaneous white adipose tissue biopsies from the periumbilical area were obtained by needle biopsies. One part of the tissue was rinsed and snap frozen in liquid nitrogen and stored at -80°C until analysis. One part was rinsed and fixed in PFA for immunohistochemistry. Details on additional analyses are provided in the paper including relevant references. |
| Outcomes | Primary and secondary outcome measures for each study are detailed at clinicaltrials.gov. |
