# Supplementary figures and images for: Impaired phosphocreatine metabolism in white adipocytes promotes inflammation
Source: Nat Metab. 2022 Feb 14;4(2):190–202. doi: 10.1038/s42255-022-00525-9 (PMC8885409; doi:10.1038/s42255-022-00525-9)

Fig. 1C

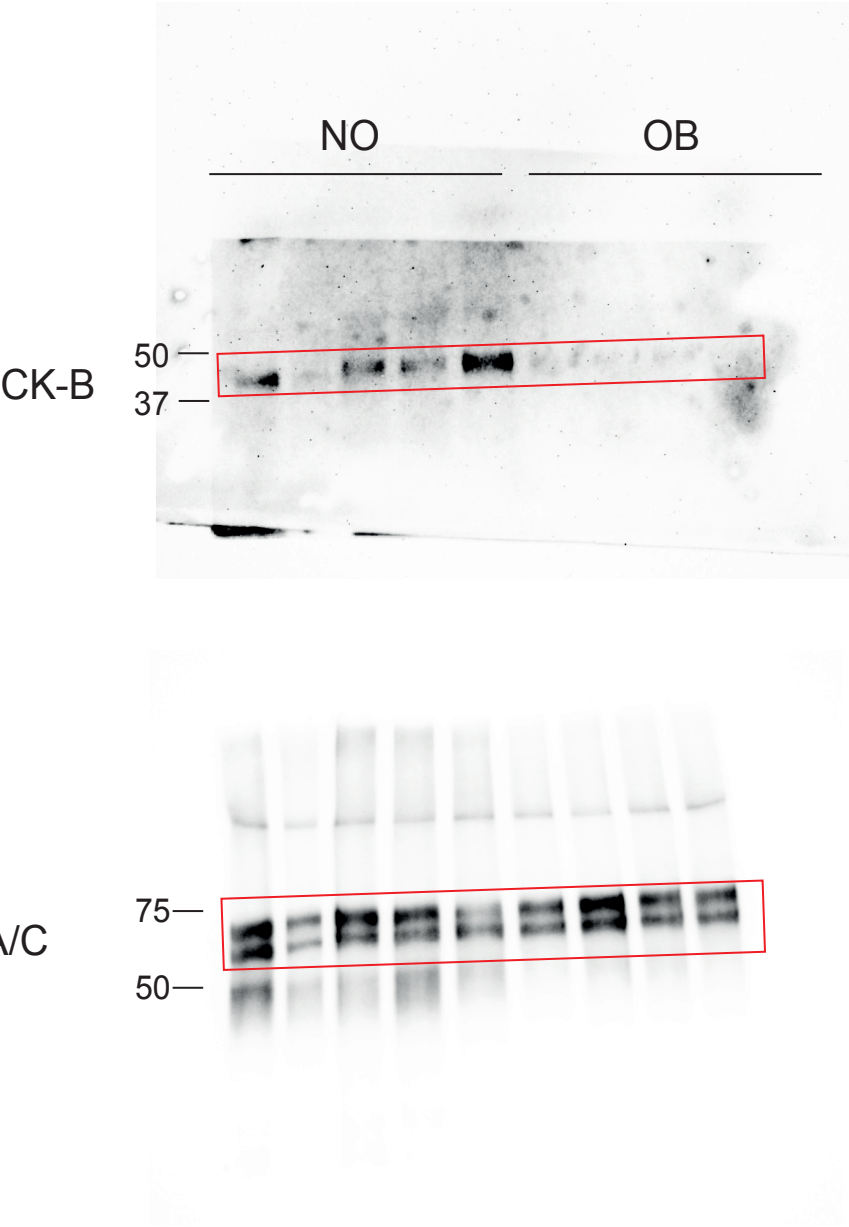

Supplement: Source Data Fig. 1 — Unprocessed western blots for Fig. 1. [file 42255_2022_525_MOESM3_ESM.pdf]

Fig. 2D

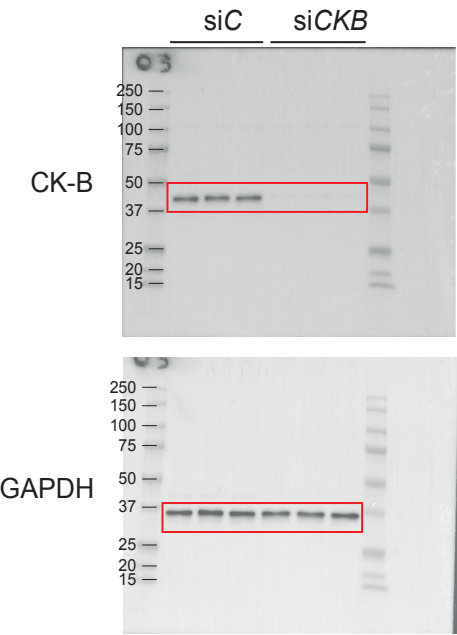

Fig. 2G

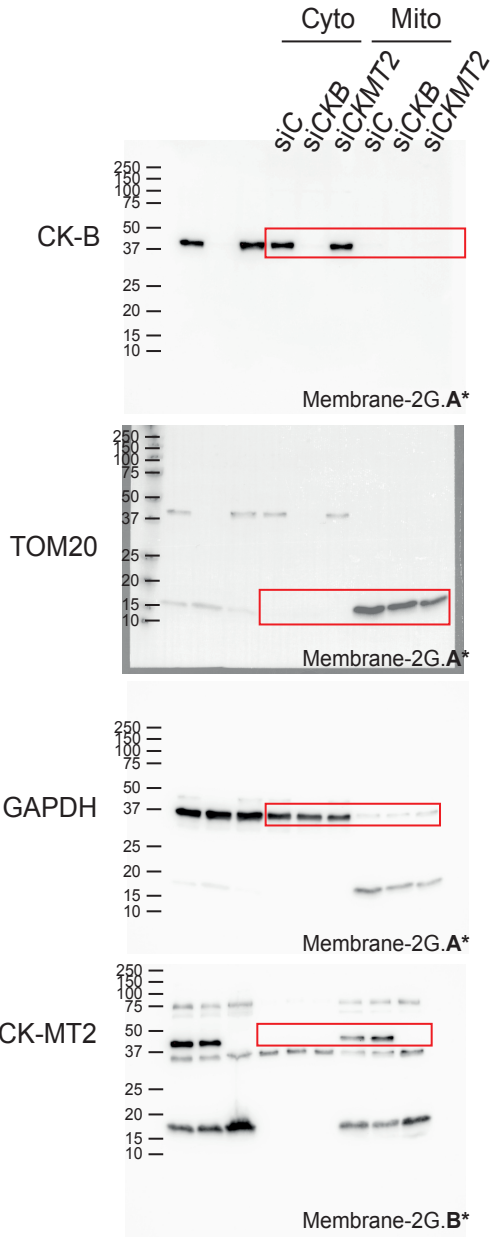

\*Lysates were subdivided in equal amounts and loaded on two separate gels.

Supplement: Source Data Fig. 2 — Unprocessed western blots for Fig. 2. [file 42255_2022_525_MOESM4_ESM.pdf]

Fig. 7B

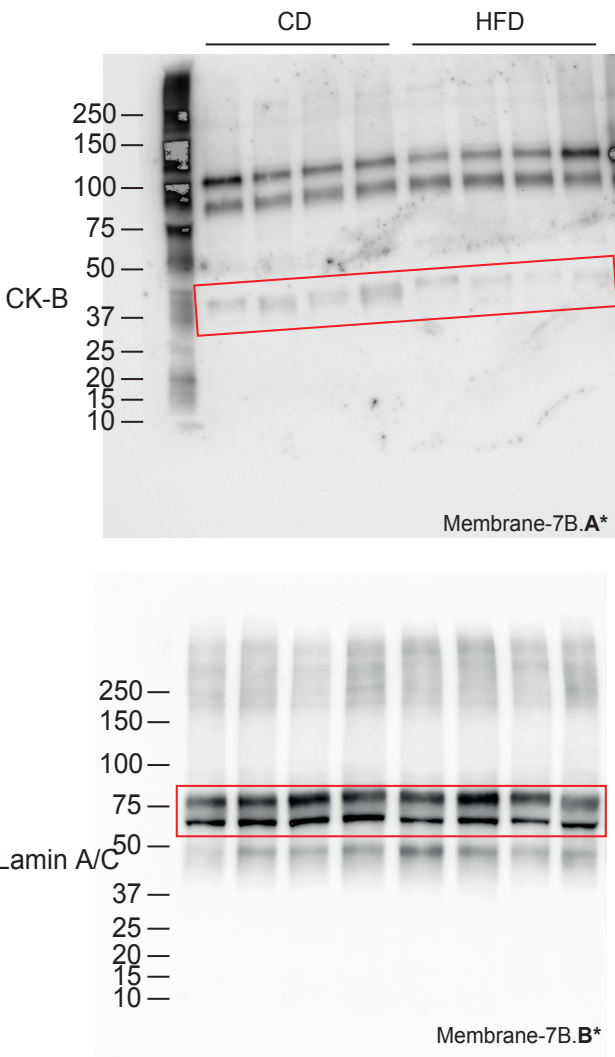

Fig. 7G

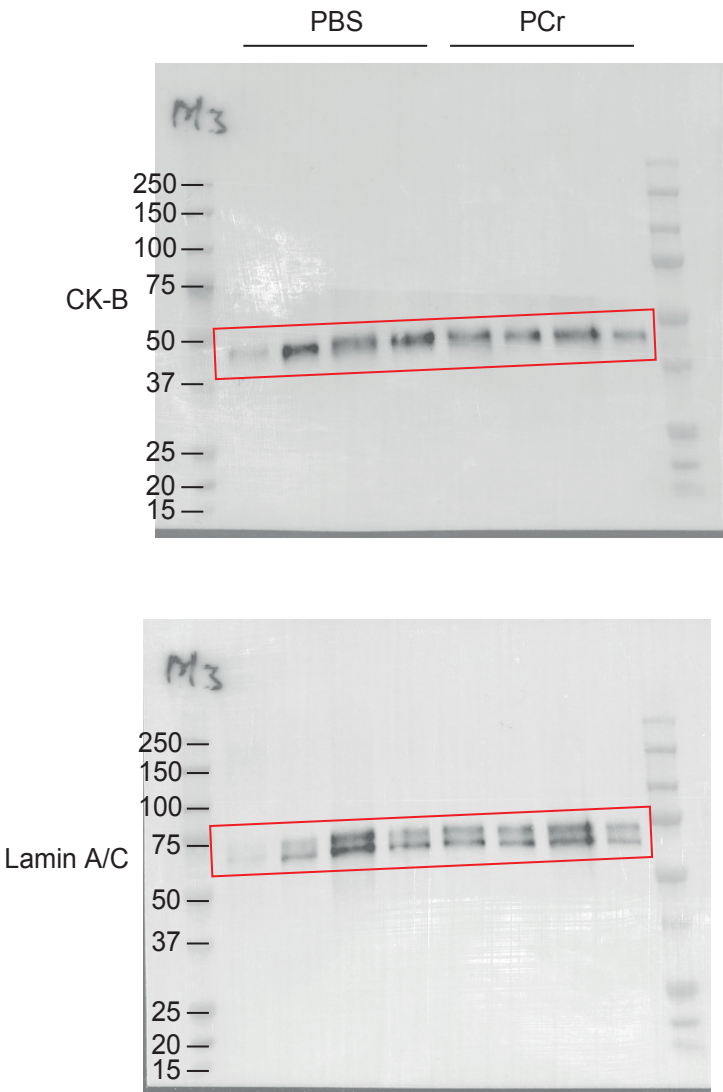

\*Lysates were subdivided in equal amounts and loaded on two separate gels.

Supplement: Source Data Fig. 7 — Unprocessed western blots for Fig. 7. [file 42255_2022_525_MOESM6_ESM.pdf]
