# Supplementary material for: Impaired phosphocreatine metabolism in white adipocytes promotes inflammation
Source: Nat Metab. 2022 Feb 14;4(2):190–202. doi: 10.1038/s42255-022-00525-9 (PMC8885409; doi:10.1038/s42255-022-00525-9)
Supplement: Source Data Fig. 6 — Unprocessed western blots for Fig. 6. [file 42255_2022_525_MOESM5_ESM.pdf]

Unmodified blots Figure 6

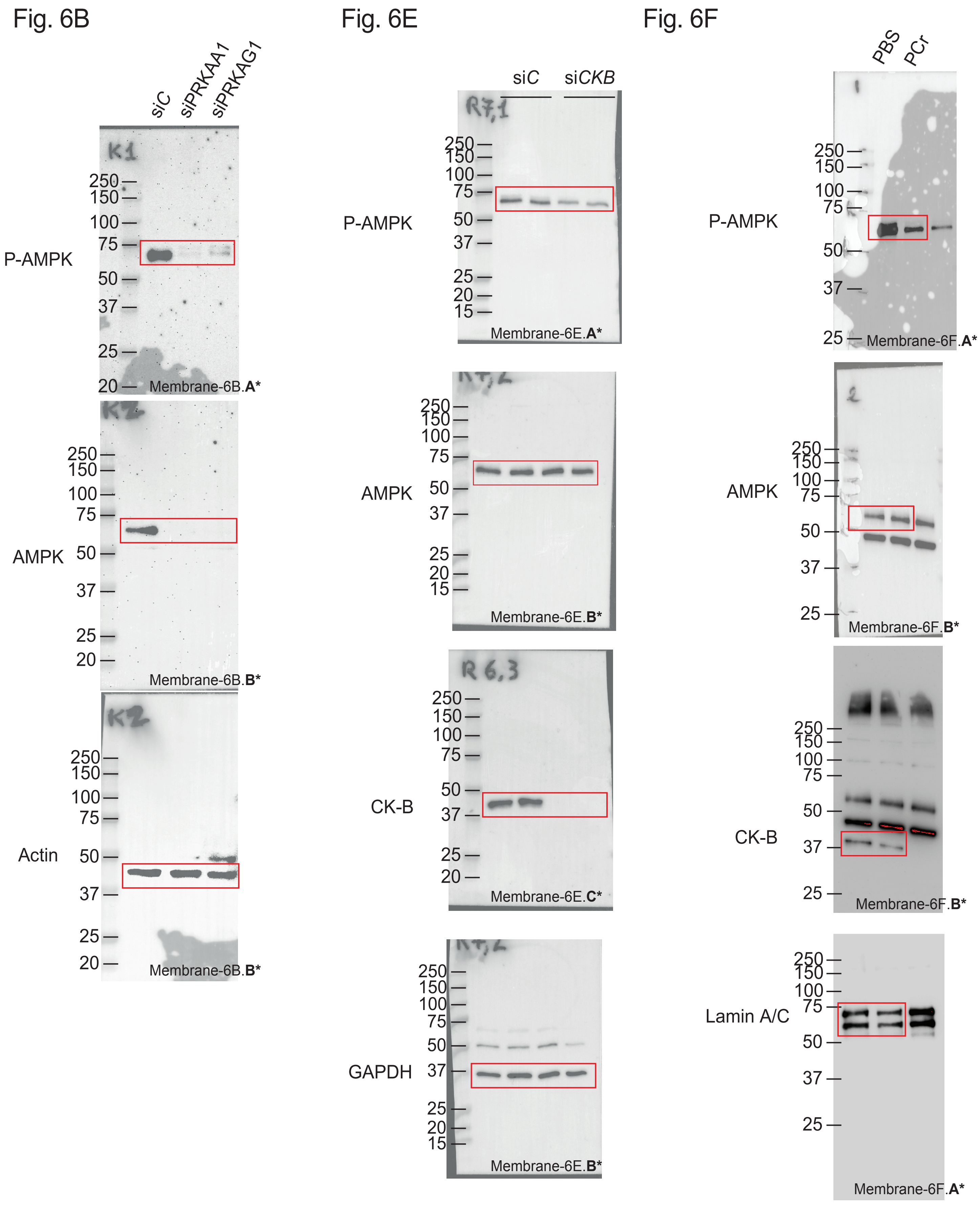

*Lysates were subdivided in equal amounts and loaded on two separate gels.
