# Supplementary material for: Impaired phosphocreatine metabolism in white adipocytes promotes inflammation
Source: Nat Metab. 2022 Feb 14;4(2):190–202. doi: 10.1038/s42255-022-00525-9 (PMC8885409; doi:10.1038/s42255-022-00525-9)
Supplement: Source Data Extended Data Fig. 3 — Unprocessed western blots for Extended Data Fig. 3. [file 42255_2022_525_MOESM7_ESM.pdf]

Unmodified blots ED Figure 3

ED Fig. 3D

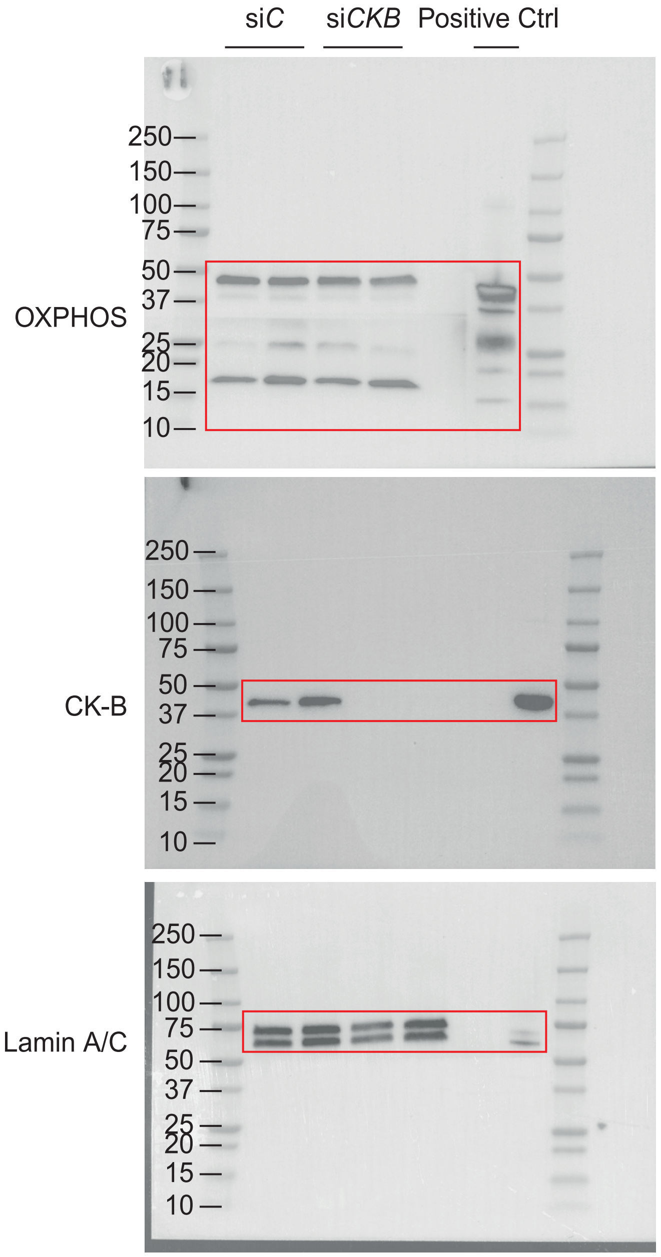

ED Fig. 3E

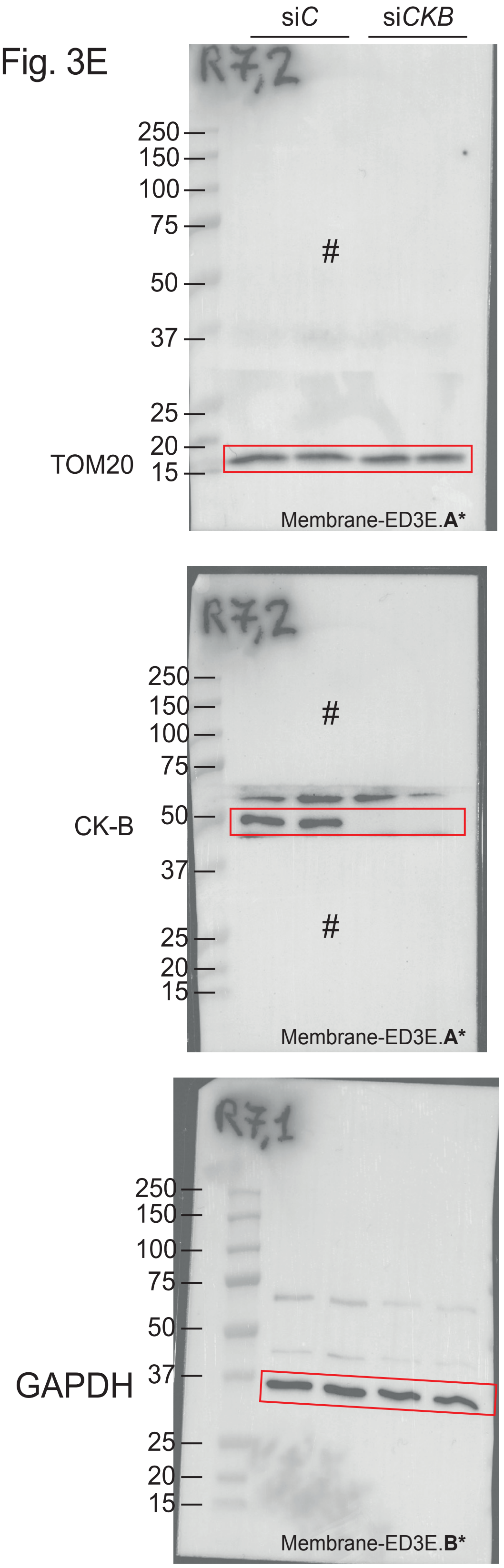

*Lysates were subdivided in equal amounts and loaded on two separate gels.

#Membrane covered in this area (instead of cropping) to allow longer exposure times for the band of interest.
